# A robust and efficient statistical method for genetic association studies using case and control samples from multiple cohorts

**DOI:** 10.1186/1471-2164-14-88

**Published:** 2013-02-08

**Authors:** Minghui Wang, Lin Wang, Ning Jiang, Tianye Jia, Zewei Luo

**Affiliations:** 1 Department of Biostatistics and Computational Biology, State Key Laboratory of Genetic Engineering, School of Life Sciences, Fudan University, 200433, Shanghai, China; 2 School of Biosciences, The University of Birmingham, Edgbaston, B15 2TT, Birmingham, UK; 3 BioSS Unit, Scottish Crop Research Institute, Invergowrie, DD2 5DA, Dundee, Scotland, UK

**Keywords:** Case and control samples, Genome-wide association study, Linkage disequilibrium, Multiple cohorts, Parkinson’s disease, Robust statistical approach

## Abstract

**Background:**

The theoretical basis of genome-wide association studies (GWAS) is statistical inference of linkage disequilibrium (LD) between any polymorphic marker and a putative disease locus. Most methods widely implemented for such analyses are vulnerable to several key demographic factors and deliver a poor statistical power for detecting genuine associations and also a high false positive rate. Here, we present a likelihood-based statistical approach that accounts properly for non-random nature of case–control samples in regard of genotypic distribution at the loci in populations under study and confers flexibility to test for genetic association in presence of different confounding factors such as population structure, non-randomness of samples etc.

**Results:**

We implemented this novel method together with several popular methods in the literature of GWAS, to re-analyze recently published Parkinson’s disease (PD) case–control samples. The real data analysis and computer simulation show that the new method confers not only significantly improved statistical power for detecting the associations but also robustness to the difficulties stemmed from non-randomly sampling and genetic structures when compared to its rivals. In particular, the new method detected 44 significant SNPs within 25 chromosomal regions of size < 1 Mb but only 6 SNPs in two of these regions were previously detected by the trend test based methods. It discovered two SNPs located 1.18 Mb and 0.18 Mb from the PD candidates, *FGF20* and *PARK8*, without invoking false positive risk.

**Conclusions:**

We developed a novel likelihood-based method which provides adequate estimation of LD and other population model parameters by using case and control samples, the ease in integration of these samples from multiple genetically divergent populations and thus confers statistically robust and powerful analyses of GWAS. On basis of simulation studies and analysis of real datasets, we demonstrated significant improvement of the new method over the non-parametric trend test, which is the most popularly implemented in the literature of GWAS.

## Background

Rapid advancement in high-throughput sequencing techniques has greatly inspired the wave of genome-wide association studies (GWAS) to unravel the genetic basis underlying complex traits in plants, animals and humans [1-3]. The theoretical kernel of these genetic association studies is statistical inference of linkage disequilibrium (LD) between a tested polymorphic marker locus and a putative trait locus in the population of interest. A review of the rich literature has revealed that a major challenge to association studies lies in the high level of vulnerability of the LD based analyses to several demographic factors, the most prominent among which is the population stratification. It has been well documented that use of samples collected from the population with genetic structure may result in both false positive and false negative inferences of association [4]. Tremendous efforts have been invested to tackle the problem through either predicting genetic structure in the population under study [5] and incorporating this information into the association analysis [6] or adjusting the test statistic through so called genomic control [7].

In contrast to the problem raised from population stratification, the consequences of using non-random samples in association studies are usually neglected. We recently investigated the effect of using non-random samples in LD analyses and observed that estimates of LD parameters can be severely biased and that the statistical power for testing their significance substantially reduced [8]. In practice, the sampling schemes of many genetic association studies involve various types of selection and thus the samples collected are no longer random representations of the corresponding populations. A typical example is the ‘case–control sample’ used in many association studies of human diseases. In such instances, the frequencies of some disease genotypes are artificially inflated relative to the population frequencies so as to ensure sufficient representation of those genotypes carrying a rare allele. Although approaches have been developed to model and analyze ‘case-control’ samples, they are usually based on nonparametric statistical tests such as χ^2^ or trend tests etc. and rarely account for the biases described above [9]. Such approaches are statistically less sophisticated and often not robust in the presence of these influences, exposing the corresponding analyses to the risk of false positive and/or negative inferences of genetic association. We present here a novel likelihood-based statistical framework that confers improved robustness in estimation of model parameters to non-randomness of samples and thus a more powerful statistical test of LD in the presence or the absence of genetic structure. We demonstrate the improved robustness and statistical power by re-analyzing the recently published Parkinson’s disease (PD) case and control datasets [2]. In addition, we illustrate the statistical properties of the method by computer simulation study.

## Methods

We consider case and control samples from *k* genetically divergent populations, of which the *i*th population contributes a proportion of the cases (*r*_*i*_) and a proportion of the controls (*s*_*i*_). In fact, any association study virtually tests for significance of LD between two polymorphic loci: a putative disease locus and a genetic marker locus which is devoid of effect on the disease incidence. The LD coefficient *D*^(*i*)^ measures the level of association where the superscript refers to the *i*th population. Let *A* and *a* denote by the two alleles at the disease locus, and *M* and *m* by the two alleles at the marker. Let *p*^(*i*)^ and *q*^(*i*)^ be the allele frequencies at the marker and disease loci respectively in the *i*th population. Most association studies using case and control samples virtually test for the disequilibrium through testing for significance of difference in frequency of the marker allele between the case and control groups. In the present notations, the difference in marker allele frequency has a form of

(1) $$\begin{array}{cc} \Delta p_{M} & =p_{M}^{\text{case}}-p_{M}^{\text{control}}\\ & =\sum_{i=1}^{k}\left\{r_{i}\left(p_{M|a}^{\left(i\right)\text{case}}q_{a}^{\left(i\right)\text{case}}+p_{M|A}^{\left(i\right)\text{case}}q_{A}^{\left(i\right)\text{case}}\right)\right)\\ & \;\left(-s_{i}\left(p_{M|a}^{\left(i\right)\text{control}}q_{a}^{\left(i\right)\text{control}}+p_{M|A}^{\left(i\right)\text{control}}q_{A}^{\left(i\right)\text{control}}\right)\right\}\\ & =\sum_{i=1}^{k}\left[r_{i}\left\{p_{M|a}^{\left(i\right)}\left(1-q_{A}^{\left(i\right)\text{case}}\right)+p_{M|A}^{\left(i\right)}q_{A}^{\left(i\right)\text{case}}\right\}\right)\\ & \;\left(-s_{i}\left\{p_{M|a}^{\left(i\right)}\left(1-q_{A}^{\left(i\right)\text{control}}\right)+p_{M|A}^{\left(i\right)}q_{A}^{\left(i\right)\text{control}}\right\}\right]\\ & =\sum_{i=1}^{k}\left\{\left(r_{i}-s_{i}\right)p_{M|a}^{\left(i\right)}+\left(p_{M|A}^{\left(i\right)}-p_{M|a}^{\left(i\right)}\right)\right)\\ & \;\left(\left(r_{i}q_{A}^{\left(i\right)\text{case}}-s_{i}q_{A}^{\left(i\right)\text{control}}\right)\right\}\\ & =\sum_{i=1}^{k}\left[\left(r_{i}-s_{i}\right),p_{M}^{\left(i\right)},+,\frac{D^{\left(i\right)}}{q_{A}^{\left(i\right)}\left(1-q_{A}^{\left(i\right)}\right)},\left\{r_{i}\left(q_{A}^{\left(i\right)\text{case}}-q_{A}^{\left(i\right)}\right)\right)\right)\\ & \;\left(\left(-s_{i}\left(q_{A}^{\left(i\right)\text{control}}-q_{A}^{\left(i\right)}\right)\right\}\right] \end{array}$$

Derivation of equation (1) implies that the conditional probabilities of a marker allele given an allele at the disease locus are constant in both cases and controls across different subpopulations, that is $p_{M|a}^{\left(i\right)\mathrm{case}}=p_{M|a}^{\left(i\right)\mathrm{contrl}}=p_{M|a}^{\left(i\right)}$ and $p_{M|A}^{\left(i\right)\mathrm{case}}=p_{M|A}^{\left(i\right)\mathrm{contrl}}=p_{M|A}^{\left(i\right)}$. This is true if all the sub-populations are no longer subject to any further structure stratification. The simple algebraic formulation reveals that any association tests, which are based on comparing the marker allele frequency between cases and controls, share at least two common properties. First, the test statistic will not be zero even though the disease and marker loci are in linkage equilibrium in all subpopulations, i.e., all *D*^(*i*)^ = 0, if the allele frequency of the tested marker varies from one population to the other, i.e. $p_{M}^{\left(i\right)}\neq p_{M}^{\left(j\right)}\;\left(i\neq j\right)$, suggesting the risk of making false positive inference of LD if the case and control samples are collected from genetically divergent populations. Secondly, the efficiency of the association test can be greatly influenced by the sampling scheme of the cases and controls as characterized by the term $r_{i}(q_{A}^{\left(i\right)\mathrm{case}}-q_{A}^{\left(i\right)})-s_{i}(q_{A}^{\left(i\right)\mathrm{control}}-q_{A}^{\left(i\right)})$ in equation (1) as we and others have previously demonstrated [4,8].

**Method 1** proposed in the present study uses information from the conditional probability distribution of genotypes at the disease locus given any genotype at the tested marker (Table 1), and develops a likelihood-based framework to infer the LD parameter. On the one hand, this provides a natural way to incorporate samples from multiple resources into the association study, and thus effectively removes the risk of false positive inference due to the genetic structure. On the other, the way, by which the likelihood analysis is formulated on the conditional probability distributions, has virtually avoided the influence of any non-randomness in sample and thus confers robustness to the sampling influence. To compare the method with one of the most popularly used approaches in the current literature of genetic association study, the Armitage’s trend test [9] was implemented to analyze the same datasets in parallel to **Method 1**, and we denote the Armitage’s trend test by **Method 3**. Based on the form of equation (1), we proposed **Method 2**, which is a modified version of the Armitage test (**Method 3** here) by removing the population structure specific term

$$\sum_{i=1}^{k}\left(r_{i}-s_{i}\right)p_{M}^{\left(i\right)}$$

from the numerator of the Armitage’s trend test statistic. All three methods are detailed in the following text.

**Table 1:** Conditional probability distributions

								
a.								
	*AA*			*Aa*			*aa*	
*MM*	*Mm*	*mm*	*MM*	*Mm*	*mm*	*MM*	*Mm*	*mm*
*g*_*11*_	*g*_*12*_	*g*_*13*_	*g*_*21*_	*g*_*22*_	*g*_*23*_	*g*_*31*_	*g*_*32*_	*g*_*33*_
*Q*^2^	2*Q*(1-*Q*)	(1-*Q*)^2^	*QR*	*Q* + *R*-2*QR*	(1-*Q*)(1-*R*)	*R*^2^	2*R*(1-*R*)	(1-*R*)^2^
where *Q* = *p* + *D*/*q* and *R* = *p* - *D*/(1-*q*)								
b.								
	*MM*			*Mm*			*mm*	
*AA*	*Aa*	*aa*	*AA*	*Aa*	*aa*	*AA*	*Aa*	*aa*
*h*_*11*_	*h*_*12*_	*h*_*13*_	*h*_*21*_	*h*_*22*_	*h*_*23*_	*h*_*31*_	*h*_*32*_	*h*_*33*_
*Q*^2^	2*Q*(1-*Q*)	(1-*Q*)^2^	*QR*	*Q* + *R*-2*QR*	(1-*Q*)(1-*R*)	*R*^2^	2*R*(1-*R*)	(1-*R*)^2^
where *Q* = *q* + *D*/*p* and *R* = *q* - *D*/(1-*p*)								
c.								
				Cases			Controls	
			*MM*	*Mm*	*mm*	*MM*	*Mm*	*mm*
*AA*			*f*_*1*_ × g_*11*_	*f*_*1*_ × g_*12*_	*f*_*1*_ × g_*13*_	(1-*f*_*1*_) × g_*11*_	(1-*f*_*1*_) × g_*12*_	(1-*f*_*1*_) × g_*13*_
*Aa*			*f*_*2*_ × g_*21*_	*f*_*2*_ × g_*22*_	*f*_*2*_ × g_*23*_	(1-*f*_*2*_) × g_*21*_	(1-*f*_*2*_) × g_*22*_	(1-*f*_*2*_) × g_*23*_
*aa*			*f*_*3*_ × g_*31*_	*f*_*3*_ × g_*32*_	*f*_*3*_ × g_*33*_	(1-*f*_*3*_) × g_*31*_	(1-*f*_*3*_) × g_*32*_	(1-*f*_*3*_) × g_*33*_
*#observed*			*n*_*11*_	*n*_*12*_	*n*_*13*_	*n*_*21*_	*n*_*22*_	*n*_*23*_

Conditional probability distribution of (a) marker genotypes on a given disease genotype, (b) disease genotypes on a given marker genotype and (c) marker genotypes given a genotype at the disease locus under the penetrance model of the disease gene in case/control samples. f_i_ is the penetrance that an individual in the population is affected with disease given its genotype at the disease locus is i (i = 1, 2 and 3 for genotypes AA, Aa and aa respectively).

In **Method 1**, we first consider a case–control sample of size *n* collected only from a single randomly mating population. The method focuses on gene segregation at a marker locus and a putative disease locus in this population. There are two alleles, *M* and *m*, segregating at the marker locus and two alleles, *A* and *a*, at the disease locus. For simplicity but without loss of generality, *A* is assigned to be the disease causing allele. The population genetic parameters characterising genotypic distribution of genotypes at the marker and disease loci include *p* (or *q*), frequency of marker allele *M* (or the disease allele *A*), and *D*, the coefficient of LD between genes at the two loci. Distribution of genotypes at the two loci can be expressed in terms of the population genetic parameters. Let *g*_*ij*_ = Pr{*Y* = *j* | *X* = *i*} be the conditional probability of marker genotype Y = *j* (*j* = 1, 2 and 3 for marker genotypes *MM*, *Mm* and *mm* respectively) given the disease genotype X = *i* (*i* = 1, 2, 3 for the disease genotypes *AA*, *Aa* and *aa* accordingly). Let *h*_*ij*_ = Pr{*X* = *i* | *Y* = *j*} be the conditional probability of disease genotype X = *i* given the marker genotype Y = *j*. These conditional probability distributions can be expressed in terms of the population genetic parameters *p*, *q* and *D* as given in Table 1a and 1b respectively.

The cases and controls collected from the population can be classified according to their genotypes at marker loci, while the sample size *n* is equal to sum of *n*_*ij*_ representing the number of individuals with *j*th marker genotype (*j* = 1, 2 and 3) in cases (*i* = 1) or controls (*i* =2). Table 1c illustrates the conditional probability distribution of genotypes at the disease locus for any given genotype at the marker locus among cases or controls. It can be seen that the conditional probability distribution is a function of the penetrance parameters that characterize the inheritance of the disease genes as well as the population genetic parameters. Table 1c is derived from Table 1a by noting that each disease genotype presents a unique disease risk. The model involves a total of six parameters, leaving their estimation as a typical over-parameterization problem. To ease the problem, we fixed the penetrance parameters *f*_1-3_ to take either of values (1, 1, 0), (1, ½, 0) or (1, 0, 0), which correspond to the dominant, additive or recessive inheritance of the disease gene *A*. Our focus was on estimation of the population genetic parameters. Consequence of possible mis-specification of penetrance parameters will be evaluated through simulation study as detailed in Results section below.

The marker allele frequency *p* can be derived through an independent population survey, or, if taking controls as a random sample of the population in regard to the marker genotypes, approximately estimated from controls as $\hat{p}=\left(n_{21}+0.5n_{22}\right)/n_{2}$ , where *n*_2_ = *n*_21_ + *n*_22_ + *n*_23_. Appropriateness will be discussed for estimating frequency *p* from control subjects in Discussion. Let *N* = (*n*_11_,*n*_12_,*n*_13_,*n*_21_,*n*_22_,*n*_23_) be a vector of the observed numbers of individuals with different marker genotypes in the case and control samples. The logarithm of the likelihood function of the parameters, *q* and *D*, given the observed vector *N* and the penetrance parameters can be expressed as

(2) $$L\left(p,q,D|N,f_{1},f_{2},f_{3}\right)\propto \sum_{j=1}^{3}\left[\sum_{i=1}^{3}\left\{\left(n_{1j}w_{\mathrm{ij}}+n_{2j}v_{\mathrm{ij}}\right)\log\left(g_{\mathrm{ij}}\right)\right\}\right]\text{,}$$

where

(3) $$\begin{array}{cc} \;w_{\mathrm{ij}} & =\frac{f_{i}h_{\mathrm{ij}}}{f_{1}h_{1j}+f_{2}h_{2j}+f_{3}h_{3j}}\mathrm{and}\\ \;v_{\mathrm{ij}} & =\frac{\left(1-f_{i}\right)h_{\mathrm{ij}}}{\left(1-f_{1}\right)h_{1j}+\left(1-f_{2}\right)h_{2j}+\left(1-f_{3}\right)h_{3j}} \end{array}$$

are the conditional probabilities that any case or control individual with the *j*th marker genotype (*j* =1, 2, 3 for *MM*, *Mm* and *mm* respectively) has the *i*th genotype at the disease locus (*i* = 1, 2, 3 for *AA*, *Aa* and *aa* respectively). Two facts should be noted to the likelihood function (2). Firstly, information about the disease genotypes is missing and *n*_1*j*_*w*_*ij*_ (or *n*_2*j*_*v*_*ij*_) represents the expected number of individuals with the *i*th disease genotype and *j*th marker genotype in the case (or control) sample. Secondly, the conditional probability *h*_*ij*_ is a function of parameters *p*, *q* and *D* as given in Table 1b. The partial derivatives of the likelihood function with respect to the unknown parameters *q* and *D* led to two normal equations

(4) $$a_{6}q^{6}+a_{5}q^{5}+a_{4}q^{4}+a_{3}q^{3}+a_{2}q^{2}+a_{1}q+a_{0}=0$$

and

(5) $$b_{5}D^{5}+b_{4}D^{4}+b_{3}D^{3}+b_{2}D^{2}+b_{1}D+b_{0}=0.$$

The coefficients *a*_*i*_ (*i* = 0,1,…,6) and *b*_*i*_ (*i* = 0,1,…,5) in equations (4) and (5) were functions of the sample observations *N* = (*n*_11_,*n*_12_,*n*_13_,*n*_21_,*n*_22_,*n*_23_) and the conditional probabilities *w*_*ij*_ and *v*_*ij*_. Mathematical forms of these coefficients were derived using the computer software Mathematica [10] and listed in Additional file 1. We proposed here an EM algorithm to calculate the maximum likelihood estimates (MLEs) of parameters *q* and *D*. The algorithm starts with the estimate of marker allele frequency, $\hat{p}$, and initial guess for values of the other two model parameters, *D* and *q*. With these parameter values, the conditional probabilities *w*_*ij*_ and *v*_*ij*_ can be calculated from equation (3). This constitutes the expectation (E) step of the EM algorithm. The maximization (M) step calculates new values of the parameters by solving equations (4) and (5) respectively. It should be noted that the coefficient of the leading term in the polynomial equations (4) and (5) is a positive constant, warranting the existence of at least one real root to these equations. Although there was no analytical solution to these equations, they can be solved numerically [11]. When multiple real roots were found, we selected the one that was within the corresponding theoretical bounds (0 < *q* < 1 and/or *max*{-*pq*, -(1-*p*)(1-*q*)} ≤ *D* ≤ *min*{*p*(1-*q*), (1-*p*)*q*}) and also resulted in the highest value of the likelihood. As the E and M steps are iteratively repeated, the likelihood function increases monotonically along the sequence of the newly determined estimates of the parameters, which converge to the MLEs of the model parameters, $\hat{q}$ and $\hat{D}$

. Significance of the disequilibrium parameter *D* can be tested using the likelihood ratio (LR) test statistic given by

(6) $$\mathrm{LR}=-2\left\{L\left(\hat{p},\hat{q},D=0|N,f_{1},f_{2},f_{3}\right)-L\left(\hat{p},\hat{q},\hat{D}|N,f_{1},f_{2},f_{3}\right)\right\}$$

It is important to note that the likelihood function under the null hypothesis can be simplified to be *L*(*p*, *q*, *D* = 0|*N*, *f*_1_, *f*_2_, *f*_3_) = (*n*_11_ + *n*_21_)Log[*p*^2^] + (*n*_12_ + *n*_22_)Log[2*p*(1 - *p*)] + (*n*_13_ + *n*_23_)Log[(1 - *p*)^2^], which is a function of the vector *N* and marker allele frequency *p* only and is independent of the other parameters, *q* and *D*. Thus, the likelihood ratio test statistic can be approximated by a *χ*^2^ distribution with 2 degrees of freedom (d.f.). Under *D* = 0, the MLE of *p* is given by $\hat{p}=\left(2n_{11}+2n_{21}+n_{12}+n_{22}\right)/2n$ as expected.

When the cases and controls are collected independently from *k* subpopulations or genetic cohorts, we formulate the likelihood of the congregate case and control sample as the sum of the likelihoods for the case and control sample from each of these cohorts as given by

(7) $$\begin{array}{c} L\left(p^{\left(1\right)},p^{\left(2\right)},..,p^{\left(k\right)},q^{\left(1\right)},q^{\left(2\right)},..,q^{\left(k\right)},D^{\left(1\right)},D^{\left(2\right)},..,\right)\\ \left(D^{\left(k\right)}|N^{\left(1\right)},N^{\left(2\right)},..,N^{\left(k\right)},f_{1},f_{2},f_{3}\right)\\ =\sum_{i=1}^{k}L\left(p^{\left(i\right)},q^{\left(i\right)},D^{\left(i\right)}|N^{\left(i\right)},f_{1},f_{2},f_{3}\right) \end{array}$$

where the superscript is used to denote the parameters for each subpopulation. To calculate the above likelihood function, we proposed firstly to work out the population specific parameters *q*^(*i*)^ and *D*^(i)^ from the case and control sample of the *i*th subpopulation separately using the method described above, and then to sum up the likelihoods for all the case and control samples. Although the likelihood ratio statistic confers the flexibility to test for significance of LD in any subpopulations, we are interested here in testing for a conservative null hypothesis that there is no LD between marker and disease loci in all the subpopulations based on the ratio of the congregate likelihood with ${\hat{D}}^{\left(i\right)}$ over the likelihood with *D*^(*i*)^ = 0. This likelihood ratio test statistic was approximated by a *χ*^2^ variable with 2 *k* d.f..

**Method 2** was modified from the Armitage’s trend test [9] for genetic association using case and control samples and from our formulation of the trend test statistic when the cases and controls are collected from *k* genetically divergent populations or cohorts as demonstrated in equation (1). The Armitage’s method basically tests for association of a polymorphic genetic marker with a disease phenotype through testing for significance of the difference in allele frequency at the marker between cases and controls. In the presence of genetic structure, the difference in frequency of the marker allele *M* between the cases and controls contains a term $\sum_{i=1}^{k}\left(r_{i}-s_{i}\right)p_{M}^{\left(i\right)}$ in which *r*_*i*_ and *s*_*i*_ stand for proportion of cases and controls collected from the *i*th population and $p_{M}^{\left(i\right)}$ for frequency of the marker allele *M* in the population as shown in equation (1). We removed this term from the numerator of Armitage’s trend test statistic, adjusted the corresponding sampling variance of the difference term and proposed

(8) $$\chi_{G}^{2}=\frac{{\left\{\Delta {\hat{p}}_{M}-\sum_{i=1}^{k}\left(r_{i}-s_{i}\right){\hat{p}}_{M}^{\left(i\right)}\right\}}^{2}}{\sum_{i=1}^{k}0.5{\hat{p}}_{M}^{\left(i\right)}\left(1-{\hat{p}}_{M}^{\left(i\right)}\right)\left\{r_{i}^{2}/n_{i}^{\text{case}}+s_{i}^{2}/n_{i}^{\text{control}}+{\left(r_{i}-s_{i}\right)}^{2}/n^{\left(i\right)}\right\}}$$

to be the test statistic which follows the chi-square distribution with 1 d.f.. In equation (8), the denominator was the sampling variance of the numerator under the null hypothesis, i.e. there is no LD in either subpopulation. $n_{i}^{\mathrm{case}}$ (or $n_{i}^{\mathrm{control}}$) and *n*^(*i*)^ are the number of cases (or controls) and size of cases and controls from the *i*th population or cohort.

**Method 3** is virtually the Armitage’s trend test, which is the most commonly implemented approach in the literature of GWAS with a case and control design. The test statistic is built upon the number of genotypes, *n*_*ij*_ with *i* =1, 2 corresponding to case and control and *j* = 1, 2, 3 to three genotypes at a tested marker, and has a form of

(9) $$Y^{2}=\frac{n{\left(n\sum_{j=1}^{3}n_{1j}x_{j}-n_{1\cdot }\sum_{j=1}^{3}n_{\cdot j}x_{j}\right)}^{2}}{n_{1\cdot }n_{2\cdot }\left\{n\sum_{j=1}^{3}n_{\cdot j}x_{j}^{2}-{\left(\sum_{j=1}^{3}n_{\cdot j}x_{j}\right)}^{2}\right\}}$$

Under the null hypothesis of no association between the marker and the disease locus, this follows a *χ*^2^ distribution with 1 d.f., where $n=\sum_{i=1}^{2}\sum_{j=1}^{3}n_{\mathrm{ij}}$, $n_{i\cdot }=\sum_{j=1}^{3}n_{\mathrm{ij}}$, $n_{\cdot j}=\sum_{i=1}^{2}n_{\mathrm{ij}}$ and the trend coefficients *x*_*j*_ (*j* = 1, 2, 3) are the weights of effects of different marker genotypes on the disease trait [9]. When *x*_1-3_ take a form of (1, 1, 0), (1, 0, 0) or (2, 1, 0), the test statistic corresponds to testing for genetic association of the tested marker with a putative disease trait showing dominant, recessive, and additive genetic inheritance respectively. As demonstrated by Jackson et al. [12], the Armitage’s trend test statistic can be expressed as

(10) $$\begin{array}{cc} \;Y^{2} & =\frac{n{\left\{n\left(2n_{11}+n_{12}\right)-n_{1\cdot }\left(2n_{\cdot 1}+n_{\cdot 2}\right)\right\}}^{2}}{n_{1\cdot }n_{2\cdot }\left\{n\left(4n_{\cdot 1}+n_{\cdot 2}\right)-{\left(2n_{\cdot 1}+n_{\cdot 2}\right)}^{2}\right\}}\\ & =\frac{{\left({\hat{p}}_{M}^{\text{case}}-{\hat{p}}_{M}^{\text{control}}\right)}^{2}}{\left\{{\hat{p}}_{M}\left(1-{\hat{p}}_{M}\right)+{\hat{p}}_{\mathrm{MM}}-{\hat{p}}_{M}^{2}\}(1/2n_{1\cdot }+1/2n_{2\cdot }\right)} \end{array}$$

under an additive genetic inheritance model [9,13], where ${\hat{p}}_{M}=\left(2n_{11}+n_{12}+2n_{21}+n_{22}\right)/2n$ , ${\hat{p}}_{\mathrm{MM}}=\left(n_{11}+n_{21}\right)/n$ , ${\hat{p}}_{M}^{\text{case}}=\left(2n_{11}+n_{12}\right)/2n_{1\cdot }$ , and ${\hat{p}}_{M}^{\text{control}}=\left(2n_{21}+n_{22}\right)/2n_{2\cdot }$ . Note that the denominator in equation (10) has a term ${\hat{p}}_{\mathrm{MM}}-{\hat{p}}_{M}^{2}$ , which is zero when the case–control sample is in Hardy-Weinberg Equilibrium (HWE). This term is proposed to be a correction for bias in variance estimation when there is departure from HWE due to several factors including population structure [13]. However, there is no such a correction term under dominant and recessive models.

### Re-analysis of the Parkinson’s disease datasets

We implemented the three methods described above to re-analyze the PD dataset which was recently published by Simon-Sanchez et al. [2]. The study carried out a genome wide screen for genetic variants predisposing susceptibility to the PD through a two-stage case–control design. In stage I, 4,005 individuals (971 cases and 3,034 cases) recruited from the United States and 1,686 individuals (742 cases and 944 cases) recruited from Germany were genotyped at 507,861 SNPs using Infinium BeadChips of which 463,185 SNPs with genotyping call rate larger than 95%, minor allele frequency (MAF) above 0.05 and no departure from HWE (p > 0.01) were remained [2]. Because estimates of allele frequency from a small sample may vary greatly, we further excluded those markers, at which there were less than five individuals for any genotype, from further analysis. After this quality control, a total of 447,270 SNPs were used in the present study. Principal component analysis (PCA) from genotype data was carried out to investigate the population structure for the stage I dataset by using program GCTA [14]. In stage II, which was designed as a confirmation stage, 3,392, 3,223 and 1,319 individuals were recruited from three different cohorts: the USA (1,473 cases and 1,919 controls), Germany (1,074 cases and 2,149 controls) and the UK (814 cases and 505 controls) respectively. All 7,934 individuals were genotyped for 345 SNPs which showed significant associations in analysis with stage I dataset. After applying the same quality check on the SNP data, two SNPs were excluded from the present study. The genetic association for each SNP marker was evaluated by Armitage’s trend test (**Method 3** here) and the genome-wide significance level was determined by the Bonferroni correction for the probability of an overall type I error at 5%.

### Simulation model and method

To investigate statistical properties and limitations of the method developed in the present study, we considered three schemes for sampling cases and controls from computer simulated randomly mating populations. In the first two sampling schemes, scheme A and B, we fixed the penetrance parameters *f*_1-3_ for genotypes at the disease locus to be (1, ½, 0), while in the third sampling scheme, scheme C, mild penetrance parameters (i.e. *f*_1-3_ < 1) were used. Sampling schemes A and C collected cases and controls from a single population, while scheme B sampled individuals from two genetically divergent populations with regard to a tested marker and a putative disease locus. A simulated population in the present study was fully characterized by a set of population genetic parameters, *p*, *q* and *D* (frequencies of alleles *M* and *A* at a marker locus and a disease locus respectively and the coefficient of LD between the two loci), and quantitative genetic parameters, *f*_1_, *f*_2_ and *f*_3_ (penetrance of three genotypes at the disease locus). For a given set of these parameters, genotype of a case or control subject at both the marker and disease loci was generated by randomly sampling from the conditional probability distribution given in Table 1c. The sampling process continued until the required number of cases or controls was obtained. The computer programs implementing the simulation were described and modified in Luo [15] and Wang et al. [8].

## Results

### Re-analysis of the Parkinson’s disease datasets

**Figure 1:**
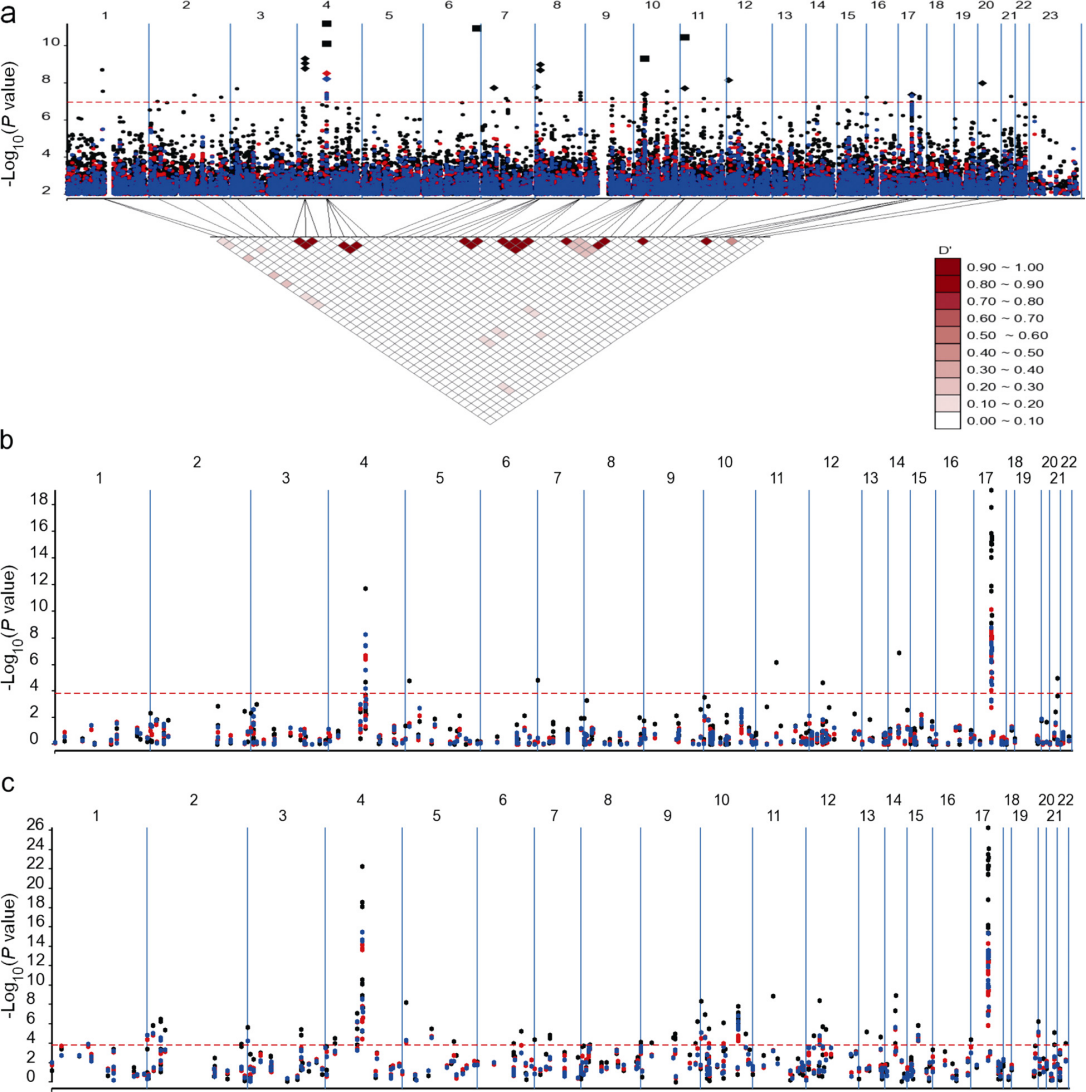
**Genome-wide association scan.** Graphic presenting association results from (**a**) stage I, (**b**) stage II and (**c**) two-stage combined case and control samples. The analysis with each of the three datasets was done using Method 1 (black circles), 2 (red circles) and 3 (blue circles) accordingly. The red horizontal dashed lines indicate the Bonferroni significance threshold of *P* value 1.1 × 10^-7^ (**a**) and 1.5 × 10^-4^ (**b** and **c**). The triangle at the bottom of (**a**) is the estimated linkage disequilibrium structure for the 44 most significant SNPs listed in Table 2. The diamonds and squares in (**a**) illustrates the SNPs at which the bootstrap posterior probability for genetic association are either > 80% or within 60 ~ 80%.

To assess the population structure in the stage I dataset, PCA was carried out using whole-genome genotype data and illustrated in Additional file 2. The analysis revealed genetic structure between the US and German individuals. Figure 1a-c illustrate distributions of the logarithmic significance levels (*P*) of genetic association tests across the 23 human chromosomes using the three PD case and control SNP datasets from the stage I, stage II and stage I and II combined, respectively. We analyzed each of the datasets using the three methods described above. For Method 1, the associations were detected under additive genetic inheritance mode with *f*_1-3_ = 1, ½, 0. It can be seen from the stage I data analysis (Figure 1a) that **Method 1** developed in the present study (black labels) detected 44 significant SNPs, which are distributed across 25 chromosomal regions of size < 1 Mb (Table 2). **Methods 2** and **3** detected significant SNPs in only two (4q21 and 17q21) of the 25 regions at the same Bonferroni corrected significance threshold (*P* ≤ 1.1 × 10^-7^). No extra significant association was detected by Method 2 or 3 outside the 25 regions screened by Method 1. To explore genetic dependence among the 44 significant SNPs detected using **Method 1**, we calculated the coefficient of LD between all SNP pairs using an approach that accounts properly for the case and control sampling scheme [8]. The disequilibrium structure illustrated at the bottom of Figure 1a shows that the significant SNPs are not associated with each other across the different regions (maximum disequilibrium measured by *r*^2^ is 0.002 between different chromosome regions), removing the concern that the detected SNP-disease associations might be due to autocorrelation in genotypic distribution among the SNPs between these regions. In particular, **Method 1** uniquely detected six SNPs on chromosome region 10p11.21, of which, rs7923172 and rs4934704 locate at the introns of gene *CUL2*. The *CUL2* gene encodes a protein of the E3 ubiquitin ligase complex [16]. The fact that another PD susceptible gene *PARK2* also encodes a parkin protein in the same complex [17] suggests that *CUL2* could be a newly detected PD candidate gene. Moreover, **Method 1** detected three SNPs on chromosome region 8p22 (the most significant *P* = 9.9 × 10^-10^ at rs2736050), which were only 1.2 Mb apart from a previously reported PD susceptible gene *FGF20*. *FGF20* and *SNCA* have previously been reported to be synergistically associated with PD [18].

**Table 2:** Summary of top associations from stage I dataset

**Locus**	**SNP name**	Dist(kb) *	***P *value**			**BPP (%)**		
			**M 1**	**M 2**	**M 3**	**M 1**	**M 2**	**M 3**
1p13.2-13.3	rs17654531	-	1.9 × 10^-9^	3.2 × 10^-6^	1.2 × 10^-5^	37	22	14
	rs10857899	328	2.7 × 10^-8^	3.1 × 10^-6^	3.1 × 10^-6^	57	25	27
2p23.3	rs7564397	-	9.7 × 10^-8^	0.013	0.033	55	0	0
2q21.2	rs1474406	-	4.3 × 10^-8^	2.3 × 10^-3^	0.001	57	1	3
2q36.1	rs1447108	-	5.5 × 10^-8^	2.5 × 10^-4^	4.4 × 10^-4^	59	4	3
3p24.3	rs1605527	-	2.0 × 10^-8^	1.0 × 10^-4^	9.4 × 10^-5^	53	9	10
4p15.2	rs6820719	-	1.6 × 10^-9^	0.23	0.30	74	0	0
	rs7676830	23	8.6 × 10^-10^	0.12	0.15	77	0	0
	rs12649499	11	4.8 × 10^-10^	0.20	0.26	77	0	0
4q21	rs11931074	-	3.9 × 10^-8^	5.1 × 10^-8^	4.8 × 10^-8^	56	54	54
	rs356220	2	7.7 × 10^-11^	3.4 × 10^-8^	7.0 × 10^-8^	81	56	52
	rs3857059	34	5.3 × 10^-8^	4.0 × 10^-8^	3.6 × 10^-8^	56	55	56
	rs2736990	3	6.3 × 10^-12^	2.9 × 10^-9^	5.7 × 10^-9^	88	71	67
6q27	rs2072638	-	1.1 × 10^-11^	0.014	0.012	86	0	0
7p14-p13	rs859522	-	1.8 × 10^-8^	9.7 × 10^-6^	3.4 × 10^-5^	62	21	14
7q21	rs3779331	-	6.6 × 10^-8^	0.028	0.01	56	0	0
7q21.11	rs10246477	-	9.3 × 10^-8^	2.3 × 10^-5^	5.3 × 10^-5^	56	13	10
8p23.2	rs7013027	-	5.8 × 10^-8^	4.3 × 10^-6^	1.9 × 10^-6^	56	23	29
	rs4875773	63	1.6 × 10^-8^	0.02	0.044	63	0	0
8p22	rs7828611	-	8.4 × 10^-8^	1.2 × 10^-4^	6.2 × 10^-4^	55	6	3
	rs2736050	1	9.9 × 10^-10^	1.0 × 10^-5^	2.0 × 10^-4^	74	18	5
	rs2009817	3	2.0 × 10^-9^	1.3 × 10^-5^	2.1 × 10^-4^	72	16	5
8q24.23-24.3	rs4556079	-	4.8 × 10^-8^	5.0 × 10^-6^	4.8 × 10^-6^	60	20	22
	rs11781101	14	7.3 × 10^-8^	5.4 × 10^-6^	5.3 × 10^-6^	56	21	22
	rs7004938	12	3.1 × 10^-8^	3.0 × 10^-6^	3.0 × 10^-6^	59	24	25
	rs11783351	1	7.7 × 10^-8^	5.0 × 10^-6^	5.5 × 10^-6^	53	21	21
9q21.31	rs2378554	-	6.6 × 10^-8^	2.0 × 10^-6^	2.9 × 10^-5^	54	29	13
10p11.21	rs2492448	-	3.8 × 10^-8^	1.6 × 10^-6^	3.8 × 10^-6^	61	29	24
	rs11591754	12	4.8 × 10^-10^	2.5 × 10^-7^	1.7 × 10^-6^	80	43	30
	rs7923172	102	7.0 × 10^-8^	1.1 × 10^-5^	1.4 × 10^-5^	54	17	16
	rs4934704	23	7.3 × 10^-8^	1.2 × 10^-5^	1.5 × 10^-5^	54	17	16
	rs10827492	97	9.7 × 10^-8^	1.3 × 10^-5^	1.7 × 10^-5^	52	16	16
10q24.3	rs17115100	-	2.7 × 10^-8^	6.9 × 10^-6^	2.5 × 10^-5^	37	19	13
11p15.2	rs11605276	-	3.4 × 10^-11^	0.079	0.19	86	0	0
	rs10500796	45	1.9 × 10^-8^	0.18	0.30	61	0	0
11q13	rs1726764	-	6.6 × 10^-8^	0.088	0.20	53	0	0
12p13	rs10849446	-	6.7 × 10^-9^	1.1 × 10^-4^	3.7 × 10^-5^	68	6	12
16p13.3	rs11648673	-	5.5 × 10^-8^	1.3 × 10^-5^	4.8 × 10^-7^	56	15	38
17q21	rs169201	-	1.0 × 10^-7^	6.5 × 10^-6^	1.2 × 10^-7^	57	19	49
	rs199533	39	4.1 × 10^-8^	2.8 × 10^-6^	5.0 × 10^-8^	60	24	55
17q24.3	rs558076	-	6.6 × 10^-8^	1.0 × 10^-4^	2.5 × 10^-5^	57	7	14
	rs817097	42	5.0 × 10^-8^	8.1 × 10^-6^	6.2 × 10^-6^	56	18	18
20p12.1	rs6041636	-	9.9 × 10^-9^	0.16	0.24	66	0	0
21q22.3	rs2070535	-	5.0 × 10^-8^	0.060	0.096	54	0	0

Significance and bootstrap posterior probabilities (BPP) for the 44 significant SNPs detected by Method 1 (M 1) from stage I dataset. Shadowed are the regions at which the genetic association was tested by Method 2 (M2) and Method 3 (M 3) at the same significance level. *Distance (kb) from previous significant SNP in the same chromosome region.

To assess variation of the predicted genetic associations, we carried out bootstrap sampling with replacement from the stage I dataset (1,000 replicates) and calculated the empirical posterior probability at each of the 44 significant SNPs. Table 2 summarizes the significance levels (*P* values) and the bootstrap posterior probabilities (BPP) calculated for each of the three methods. BPP was calculated as the proportion of bootstraps in which the SNP of interest was detected given the empirical Bonferroni *P* value threshold of 1.1 × 10^-7^. Of the three methods tested on the stage I dataset, we find that **Method 1** confers the most powerful test for the genetic association. The BPP values calculated from repeated bootstrap samples by analysis using **Method 1** were consistently higher (Wilcoxon signed-rank test *P* value 5.8 × 10^-9^), suggesting the method is more robust to variation caused from sampling than the other two methods tested in this study. Method 2 and 3 had comparable BPP values (Wilcoxon signed-rank test *P* value 0.57) and hence similar robustness to sampling variation.

Before reporting our analysis of the stage II dataset, it is worth stressing that the 345 SNPs originally genotyped were selected only from the previous analysis using **Method 3**[2]. The dataset contains only 27 of the 44 significant SNPs identified using **Method 1** in our analysis of the stage I data. Using a Bonferroni corrected genome-wide *P* = 0.05 significant threshold (1.5 × 10^-4^), we found the SNPs located within 4q21 and 17q21 to be repeatedly detected by all the three methods in the stage II dataset, while an additional six SNPs were detected by **Method 1** to be in significant association with the disease trait at the Bonferroni genome-wide threshold (Figure 1b). The maximum *r*^2^ between the six SNPs identified only by **Method 1** was 0.0013. In particular, analysis using **Method 1** detected a significant SNP (rs11564162) within chromosome 12q12, (*P* = 2.2 × 10^-5^), located just 176 Kb from the previously identified PD candidate gene *PARK8*[19]; neither **Method 2** nor **3** detected this significant SNP. In addition, a significant SNP (rs2878172) within chromosome 14q22.2 detected by **Method 1** is only 4 Kb from the gene GCH1, which was recently found to be associated with PD through meta-analysis of multiple PD GWAS datasets and curated in the PDGene database [20]. A full list of significant SNPs detected by the three methods in the analysis of the stage II dataset are shown in Additional file 3.

When the two datasets (stage I and stage II) were combined, 90 SNPs were detected significant at the Bonferroni corrected *P* = 0.05 threshold (1.5 × 10^-4^) using **Method 1**, including all the twenty seven significant SNPs detected using the same method in stage II analysis and eight significant SNPs detected by the same method in stage I data analysis (Figure 1c and Additional file 3). The 55 SNPs undetected in individual dataset were distributed in 39 chromosomes regions (*r*^2^ between regions was less than 0.0012). The SNP marking the PD candidate gene, *PARK8*, detected in the analysis of the stage II dataset, was also repeated in analysis with the combined dataset. Query against the PDGene database [20], we found another 6 out of the 55 SNPs that had been reported to be associated with PD: rs6812193 (OR 0.89, 95% confidence interval (CI) 0.85-0.93), rs6532197 (OR 1.31, 95% CI 1.19-1.44), rs7077361 (OR 0.86, 95% CI 0.79-0.93), rs11191425 (OR 0.84, 95% 0.75-0.93), rs12413409 (OR 0.84, 95% CI 0.75-0.95) and rs1481088 (OR 1.08, 95% CI 1.01-1.16). A majority of the thirty-five significant SNPs that replicated the stage I or II analysis were detected with markedly more stringent significant levels, reflecting the increased size of the combined dataset.

**Figure 2:**
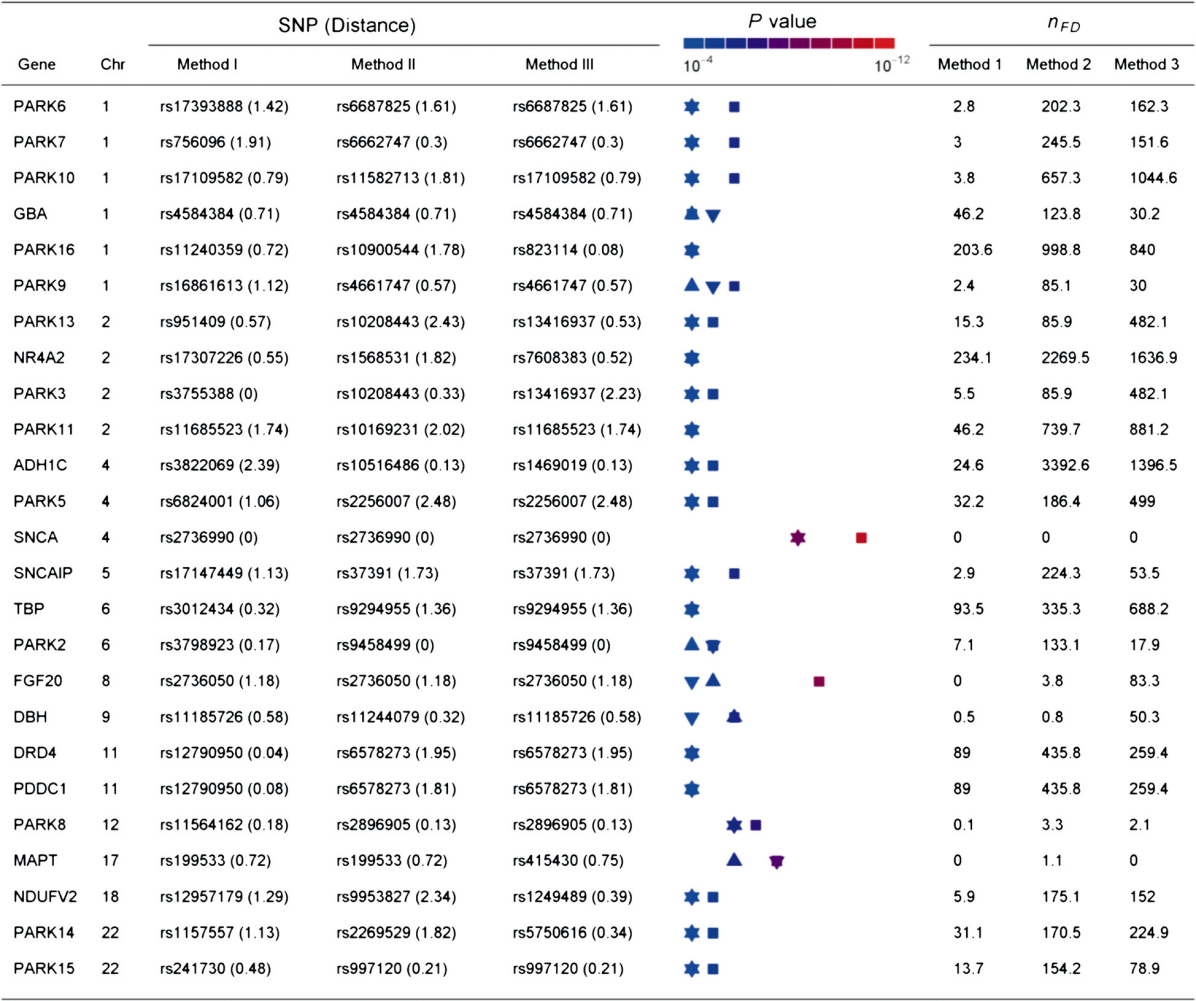
**Significance of Parkinson’s disease candidate genes.** The most significant SNP within ±2.5 Mb chromosome regions surrounding each of 25 Parkinson’s disease (PD) candidate genes. In parentheses is the physical distance (Mb) of the SNP to the corresponding PD candidate gene. *P* values are calculated from analysis of stage I dataset with Method 1 (square) and 2 (up triangle) and 3 (down triangle), and presented in the color bar depicting varying levels of significance probability. Note some data points are overlapped. *n*_*FD*_ refers as to estimate of the number of false discoveries for a given *P* value.

There have been a total of twenty five candidate genes discovered so far to predispose individuals to Parkinson’s disease (the OMIM database with entry 168600). We explored the extent to which these candidate genes can be revealed in the present genetic association study. Listed in Figure 2 are the most significant SNPs within a 2.5 Mb chromosomal region surrounding each of the 25 PD candidate genes and estimate of the number of false discoveries evaluated at the probability at which the SNP was claimed significant [21]. It can be seen that all the three methods detected the SNP, rs2736990, within the PD candidate gene *SNCA*[22] on human chromosome 4q21 as well as the SNP rs199533, just 0.72 Mb distant from the PD candidate gene *MAPT*[23] on chromosome 17q21 with negligible risk of being false positives. In addition to these, **Method 1** discovered two additional SNPs located 1.18 Mb and 0.18 Mb from the PD candidates, *FGF20*[18] and *PARK8*[19] respectively without invoking the risk of false positive. The identification of significant genetic markers close (< 1.2 Mb) to the PD genes further supports the improved efficiency of the newly developed method for genetic association study.

#### Simulation study

Table 3 presents the parameters defining 10 simulated randomly mating populations as well as means and standard deviations of estimates of the model parameters from 1,000 repeated samples of 200 cases and 200 controls. **Methods 1** and **3** were only implemented to analyze the simulation data because **Methods 2** and **3** are effectively interchangeable when the case and control samples were known to come from a single randomly mating population. When the marker and disease genes were in LE (i.e. *D* = 0 in simulated populations 1–3), marker genotype provides no information on the unobservable genotype at the disease locus and thus no estimate of disease allele frequency *q* was attempted under the circumstances. Means of the test statistic in these populations were approximately equal to 2.0 or 1.0 for **Method 1** or **3** respectively, corresponding to means of the chi-square variable with 2 or 1 degree of freedom as expected for the likelihood ratio test in **Method 1** and the chi-square test in **Method 3**. This demonstrates the adequacy of proposed distribution of the test statistic constructed in these methods under the null hypothesis and, in turn, the appropriate control of the type I error of the statistical tests. When LD was actually present (populations 4–10), **Method 1** estimated the model parameters, *q* and *D*, adequately (coefficient of variation of the square root of the mean square error is less than 0.5 for parameter estimates) and, provided a consistently higher statistical power (*ρ*) to test for significance of the disequilibrium than **Method 3**. We did also investigate the performance of **Method 1** when the disease allele showed either dominant or recessive inheritance and the simulation study demonstrates that the method predicted the model parameters well under these different genetic models (Additional file 4).

**Table 3:** Parameters and results of scheme a simulation

**Pop.**	***p***	***q***	***D***	**Method 1**				**Method 3**	
				$\hat{q}\pm s.d\text{.}$	$\hat{D}\pm s.d\text{.}$	$X_{\left[2\right]}^{2}\pm s.d\text{.}$	***ρ *(%)**	$X_{\left[1\right]}^{2}\pm s.d\text{.}$	***ρ *(%)**
1	0.5	0.5	0	-	0.004 ± 0.012	1.9 ± 2.5	6.9	1.0 ± 1.3	4.2
2	0.3	0.7	0	-	0.005 ± 0.011	2.0 ± 2.8	7.3	1.0 ± 1.4	4.5
3	0.7	0.3	0	-	0.002 ± 0.011	1.9 ± 2.7	6.7	1.0 ± 1.5	5.0
4	0.5	0.5	0.15	0.50 ± 0.05	0.148 ± 0.015	184.4 ± 42.8	100	73.3 ± 14.0	100
5	0.5	0.5	0.10	0.50 ± 0.09	0.097 ± 0.018	73.9 ± 26.5	99.7	33.3 ± 10.6	96.6
6	0.5	0.5	0.05	0.50 ± 0.20	0.043 ± 0.020	18.1 ± 12.0	36.8	8.8 ± 5.6	10.8
7	0.3	0.7	0.07	0.72 ± 0.12	0.064 ± 0.026	68.4 ± 25.4	99.6	29.6 ± 10.2	91.5
8	0.3	0.7	0.05	0.70 ± 0.15	0.047 ± 0.023	33.2 ± 17.6	77.3	15.1 ± 7.5	38.2
9	0.7	0.3	-0.07	0.28 ± 0.14	-0.062 ± 0.028	54.8 ± 23.4	96.8	26.3 ± 9.6	85.2
10	0.7	0.3	-0.05	0.31 ± 0.20	-0.042 ± 0.024	27.8 ± 15.6	66.1	13.7 ± 6.9	31.0

Population genetic parameters for 10 simulated populations and statistical inference of model parameters from 200 cases and 200 controls repeatedly sampled from the simulation populations. *p* and *q* are allelic frequencies at the marker and disease loci, *D* is the coefficient of linkage disequilibrium (LD) between the two loci. Means and standard deviations (s.d.) of the model parameters, *q* and *D*, and *χ*^2^test statistic were calculated from 1000 repeated samples. *ρ* (%) is the proportion in 1000 repeats in which the association test surpassed the threshold of P-value at 0.05 when LD is absent and the Bonferroni threshold of P-value at 5 × 10^-5^ when LD is present.

We explored the influence of using case and control samples collected from genetically divergent populations (or cohorts) on performance of the three methods. Table 4 illustrates 14 sets of simulation parameters defining the genetic structure of two randomly mating populations and the empirical power of the methods in the association test using case and control samples from these populations. The case and control samples were either taken separately or using the admixed samples of which 57% cases and 76% controls were from population 1 and the rest from population 2. These two percentage values were deliberately chosen corresponding to the constitution of cases and controls in the stage I PD dataset. When the disequilibrium was absent in either of the populations (populations 1–6), all three methods shared a low probability of making false positive inference using case and control samples from these populations separately (Table 4). When cases and controls were contributed by the two populations, the false positive rate remained at the same lower level for **Methods 1** and **2** but increased up to 25% for **Method 3**. Moreover, the increase in the false positive probability for **Method 3** was in proportion to the difference in marker allele frequency between the two contributing populations. The larger the difference, the higher the false positive probability was observed, reflecting the fact that the test statistic of this method is proportionate to the size of difference between the allele frequencies as expected from the above theoretical analysis. When the disequilibrium did truly exist in either or both simulated populations (populations 7–14), **Method 1** was able to detect it with a remarkably higher statistical power than the other two methods. In particular, when the disequilibrium had opposite signs in the two contributing populations (populations 13–14, i.e. the scenario where the disease causing gene was in association with different marker alleles in different populations), the highest detecting power was observed for **Method 1** no matter whether the case and control sample was collected from the contributing populations separately or as admixture of the populations. In contrast, the *χ*^2^ test based methods failed to detect the disequilibrium under such circumstances. These findings strongly support the improved statistical efficiency of the likelihood-based method presented here and its robustness to inherent genetic structure in the case and control samples.

**Table 4:** Parameters and results of scheme b simulation

**Pop.**	***p***^**(1)**^	***q***^**(1)**^	***D***^**(1)**^	***p***^**(2)**^	***q***^**(2)**^	***D***^**(2)**^	**Population 1**			**Population 2**			**Admixed samples**		
							**M 1**	**M 2**	**M 3**	**M 1**	**M 2**	**M 3**	**M 1**	**M 2**	**M 3**
1	0.40	0.10	0.00	0.70	0.10	0.00	0.1	0.0	0.0	1.6	0.0	0.0	1.2	0.0	25.3
2	0.45	0.10	0.00	0.70	0.10	0.00	0.0	0.0	0.0	1.0	0.0	0.0	0.6	0.0	12.6
3	0.50	0.10	0.00	0.70	0.10	0.00	0.3	0.0	0.0	1.4	0.0	0.0	1.2	0.0	3.7
4	0.55	0.10	0.00	0.70	0.10	0.00	0.2	0.0	0.0	2.1	0.0	0.0	1.1	0.0	0.9
5	0.60	0.10	0.00	0.70	0.10	0.00	0.0	0.0	0.0	1.1	0.0	0.0	1.0	0.0	0.3
6	0.65	0.10	0.00	0.70	0.10	0.00	0.1	0.1	0.1	0.9	0.0	0.0	0.5	0.0	0.0
7	0.40	0.10	0.00	0.50	0.10	0.02	0.1	0.0	0.0	94.3	44.8	45.6	91.1	2.9	50.8
8	0.45	0.10	0.00	0.50	0.10	0.02	0.0	0.0	0.0	93.4	45.7	47.2	90.8	1.4	28.4
9	0.40	0.10	0.02	0.50	0.10	0.00	99.5	93.9	94.7	1.1	0.0	0.0	99.4	70.1	90.0
10	0.45	0.10	0.02	0.50	0.10	0.00	99.7	95.4	95.5	1.1	0.0	0.0	99.3	69.3	77.4
11	0.40	0.10	0.02	0.50	0.10	0.02	99.6	95.0	95.1	93.2	43.7	45.7	100.0	99.7	100.0
12	0.45	0.10	0.02	0.50	0.10	0.02	99.6	95.2	95.6	93.1	47.5	49.0	100.0	99.7	100.0
13	0.40	0.10	0.02	0.50	0.10	-0.02	99.4	95.1	95.3	92.2	45.6	47.0	100.0	4.2	6.1
14	0.45	0.10	0.02	0.50	0.10	-0.02	99.1	93.9	94.0	94.2	45.8	47.8	100.0	3.0	1.4

Population genetic parameters defining two genetically divergent populations and empirical statistical powers of Methods 1–3 (M 1–3) for detecting significance of linkage disequilibrium between a polymorphic marker and a putative disease locus. The empirical power was calculated from 1,000 repeated samples of 1,000 cases and 1,000 controls as the proportion of the test statistic surpassing the Bonferroni threshold 5 × 10^-5^. The admixed samples were made up of 57% cases and 76% controls from Population 1 and the rest from Population 2.

While the true penetrance parameters at a disease locus are indeed unknown in practice, we proposed incorporation of the penetrance parameters with predefined values of (1, 1, 0), (1, 0, 0) or (1, ½, 0) into the analysis. This is mainly to ease the problem of over-parameterization and to set the penetrance differ among the different disease genotypes whereas the true but unknown penetrance parameters could be far less than 1 for any single locus genotype that contributes to genetic variation of common polygenic disease traits. We investigated how the use of mis-specified penetrance values would influence performance of the association tests through computer simulation. The simulation considered the scenario where the disease genotypes had very low levels of penetrance. Table 5 illustrates means and standard deviations of the test statistics and empirical power calculated from **Method 1** when true values of the penetrance parameters were used and when the penetrance parameters were set to be constant, i.e. *f*_*1-3*_ *=* (1, ½, 0) as those implemented in the above data analysis. It can be seen that mis-specification of the penetrance parameters in analysis of the simulation data with the method has not caused a marked loss of statistical power in detecting the genetic association nor led to increase in false positive inference of association when the marker and disease loci are truly in linkage equilibrium (*D* = 0). This thus removes the concern about appropriateness to use the predefined setting of disease penetrance in the method developed here.

**Table 5:** Parameters and results of scheme c simulation

**Pop.**	***p***	***q***	***D***	***f***_**1**_	***f***_**2**_	***f***_**3**_	$\hat{p}\pm s.d\text{.}$	Method 1*		Method 1**		**Method 3**	
								***χ***^**2**^ **±** ***s.d.***	**ρ (%)**	***χ***^**2**^ **±** ***s.d.***	**ρ (%)**	***χ***^**2**^ **±** ***s.d.***	**ρ (%)**
1	0.5	0.5	0	0.1	0.05	0	0.50 ± 0.02	2.0 ± 6.1	2	1.9 ± 2.9	0	0.9 ± 1.3	0
2	0.3	0.7	0	0.1	0.05	0	0.30 ± 0.02	2.2 ± 5.9	2.1	1.8 ± 2.9	0.2	1.0 ± 1.3	0
3	0.7	0.3	0	0.2	0.1	0	0.70 ± 0.02	1.5 ± 3.9	0.5	2.0 ± 2.8	0	1.0 ± 1.3	0
4	0.5	0.5	0.15	0.2	0.1	0	0.48 ± 0.02	57.8 ± 21.8	99.2	52.5 ± 19.0	98	24.1 ± 9.2	79.1
5	0.5	0.5	0.1	0.1	0	0	0.49 ± 0.02	74.5 ± 24.1	99.7	68.7 ± 20.9	99.6	35.0 ± 11.0	97.7
6	0.5	0.5	0.05	0.1	0	0	0.50 ± 0.02	20.0 ± 12.3	42.6	19.6 ± 11.9	41.5	9.3 ± 5.8	11.8
7	0.3	0.7	0.07	0.3	0.1	0	0.29 ± 0.02	16.4 ± 12.7	32.4	14.2 ± 10.9	25	6.5 ± 4.9	4.5
8	0.3	0.7	0.05	0.3	0.1	0	0.29 ± 0.02	9.5 ± 8.9	12.6	8.2 ± 7.7	8.8	3.7 ± 3.6	1
9	0.7	0.3	-0.07	0.1	0	0	0.70 ± 0.02	102.6 ± 29.7	100	93.5 ± 26.1	99.9	44.7 ± 12.0	99.6
10	0.7	0.3	-0.05	0.1	0	0	0.70 ± 0.02	53.7 ± 21.1	96.6	50.5 ± 19.3	96.2	24.0 ± 9.0	80.1

Means and standard deviations (s.d.) of estimates of empirical statistical power (ρ) and the test statistic based on 200 cases and 200 controls from 1000 repeated computer simulations. The left panel lists values of the simulation parameters and the right the estimates. ρ is estimated as proportion (%) of significant tests at the Bonferroni threshold 5 × 10^-5^ in 1000 simulations.

* when the true simulated parameters were used in the association test.

** when the penetrance parameters *f*_*1-3*_ were constantly set to be (1, ½, 0).

## Discussion

We have shown that Armitage’s trend test [9], the most popular statistical strategy implemented in the current literature of GWAS with a case–control setting, is highly vulnerable to sampling schemes and genetic structure embedded in the samples. To address this problem, we have developed a novel statistical method that is robust to these influential factors and confers a more powerful test. We have demonstrated the robustness and improved statistical power of the new method through (i) re-analysis of the large-scale SNP genotype datasets of the PD cases and controls collected from multiple geographical cohorts [2], and (ii) through computer simulation studies. The new method was able to detect a total of 44 SNPs in significant association with the disease phenotype, which distributed in 25 chromosomal regions of size < 1 Mb largely in LE. Only two of these regions are detected by the other methods under comparison. Among the newly detected significant SNPs, some are within or nearby the PD candidate genes previously reported in the literature and the rest novel discoveries. A Bootstrap-based analysis shows that the new method has consistently higher posterior probabilities at the significant SNPs than the compared methods, suggesting the remarkably improved robustness of the former to the sampling problem.

We have solved three major problems in the methodology development. Firstly, genotype at the disease locus is not observable. This has led formulation of the model parameter estimation to be built on the principles of statistical analysis with missing data [24]. Secondly, the case and control samples rather than random samples are used to infer LD between any polymorphic marker locus and a putative disease locus. Several recent researches addressed this problem and developed methods for association analysis with case and control samples [25,26]. Our method differs from them in several key aspects. The present method is developed on the basis of an explicit population genetic model which is fully characterized by population frequencies of alleles at marker and disease loci as well as the coefficient of LD between the two loci. This model enables development of a novel statistical approach for directly estimating the model parameters and in turn statistical test for significance of the genetic association is built on the estimates. Given that the accurate estimation of the disequilibrium parameter is crucial for the reliability of any LD analysis including LD-based mapping of complex genetic disease traits [27], this model based analysis may explain outperformance of the parametric approach over the existing nonparametric rivals. Moreover, we presented a simple but plausible statistical model (Equation 1) for demonstrating how the population structure embedded in the case and control samples would affect any association study which tests for significance of the difference in marker allele frequency between cases and controls such as the Armitage’s trend test and many others such as the well-known Mantel-Haenszel test. With the present notation used to derive equations (1) and (8), we are able to demonstrate that the Mantel-Haenszel test statistic has a form of

(11) $$\begin{array}{cc} \;\chi_{M}^{2}\approx & {\left\{\Delta {\hat{p}}_{M}-\sum_{i=1}^{k}\left(r_{i}-s_{i}\right){\hat{p}}_{M}^{\left(i\right)}\right\}}^{2}/\\ & \sum_{i=1}^{k}0.5\left\{\frac{n^{2}r_{i}s_{i}}{n^{\left(i\right)}\sum_{j=1}^{k}n_{j}^{\text{case}}\sum_{j=1}^{k}n_{j}^{\text{control}}}{\hat{p}}_{M}^{\left(i\right)}\left(1-{\hat{p}}_{M}^{\left(i\right)}\right)\right\} \end{array}$$

which follows a chi-square distribution with 1 d.f.. Comparison of equation (11) to equation (8) shows that the two test statistics share the same numerator, and the denominator of $\chi_{G}^{2}$ is only slightly larger than that of $\chi_{M}^{2}$. Thus, the Mantel-Haenszel test is approximately equivalent to the Method 2 which has been explored in the present study. Moreover, we demonstrated in Additional file 5 that the Mantel-Haenszel test was indeed equivalent to the widely employed logistic regression approach for analyzing stratified case and control samples such as the PD datasets. Either the present Method 2 or the Mantel-Haenszel test provides an efficient alternative to the logistic regression in testing for association using case and control samples with known stratification. Thirdly, the likelihood-based method developed in the present study confers the flexibility to fit in different fixed effects in different populations and is thus logically appropriate to integrate cases and controls collected from genetically different cohorts or populations like the PD case and control samples we thoroughly analyzed here. Although a rich literature has been available for prediction of genetic structure of a census population from random samples of the population, there is no relevant theory and method established to make the prediction from case and control samples. In the present study, we have assumed that the population origin of the case and control samples is previously known. This assumption is perfectly satisfied in many association studies, as illustrated by the PD datasets, where the cases and controls are collected from known populations or cohorts.

It needs to be pointed out that a full model involves a total of six unknown parameters and thus presents an over-parameterization problem to statistical analysis under the model. To ease the problem, we have firstly proposed to estimate the marker allele frequency, *p*, from control samples. This is largely because the likelihood function (2) is formulated in terms of two intermediate variables *Q* and *R* as described in Table 1a, and these have imposed non-linear constraints on the three parameters *p*, *q* and *D*. It is thus impractical to work out estimates of these three parameters independently and simultaneously. We explored how the proposed method to estimate *p* would affect estimation of the parameters *q* and *D* and in turn statistical power of the association test. In fact, the marker allele frequency, *p*, in the controls can be expressed in the present notation as

(12) $$\begin{array}{cc} \;p\prime & =\mathrm{Pr}(M|A)\mathrm{Pr}(A|\mathrm{controls})\\ & \;+\mathrm{Pr}(M|a)\mathrm{Pr}(a|\mathrm{controls})\\ & =p+\frac{D\left[\left(f_{2}-f_{1}\right)q+\left(f_{3}-f_{2}\right)\left(1-q\right)\right]}{1-\pi } \end{array}$$

where *π* = *f*_1_*q*^2^ + 2*f*_2_*q*(1 - *q*) + *f*_3_(1 - *q*)^2^ is the population prevalence of the disease attributed to the disease locus. The second term, i.e. the bias, will be negligible when *π* is low. To illustrate magnitude of the bias, we worked out the absolute difference |*p*’-*p*| and illustrated for a wide ranges of the population settings in Additional file 6. It is clear that the absolute value of bias will not exceed 0.05 if *π* is less than 10%. It should be stressed that the bias presented here is its largest possible value because it was calculated at the maximum value of the disequilibrium parameter *D*. In addition, we compared the rate of false positive and statistical power of **Method 1** when the true and biased values of marker allele frequency were used in analysis of simulation data under a wide range of settings. The results of the analysis summarized in Additional file 7 show that use of marker allele frequency estimates from control samples does not result in any notable difference in the false positive rate and test power from use of the true marker allele frequencies. All these thus suggest that the way we proposed to calculate the marker allele frequency will not lead to any serious influence of the method developed in the present study for its efficiency in the association test.

In spite that the population genetic model has been focused on the most prominent LD measure, *D* as defined in Table 1, there are several other scaled or standardized disequilibrium measures such as *D*’, *r*^*2*^ and some others [28], which are frequently used in the literature. The robustness and improved statistical efficiency achieved in inferring *D* will be inherent to that of the transformed versions of the parameter [8]. Although the method is developed for complex quantitative traits with discrete phenotypes, it would not involve major technical difficulty to extend the ideas and principles behind the newly developed method to cope with continuous phenotypes. Genetic heterogeneity may add extra complication to genetic control of common disease traits and is not taken into account in the present model and analysis. In presence of genetic heterogeneity, disease disposing loci may differ in different populations or cohorts. A direct and intuitive consequence of the heterogeneity would be a weakened test power because the effective sample size for detecting the marker-disease association at a test site is actually reduced when compared to the census sample size.

## Conclusions

We have developed a novel likelihood based statistical approach to model linkage disequilibrium between any genetic marker locus and a putative disease locus in a randomly matting population and to infer the disequilibrium parameter and other population genetic parameters from case and control samples from the population under a likelihood based framework. The model and likelihood based approach are implemented to re-analyze large SNP datasets of the Parkinson disease case and control samples collected from multiple human cohorts. Statistical properties and utility limitations are investigated through simulation studies. Based on the simulation data analysis and analysis with the Parkinson disease case and control sample, we demonstrate that the likelihood based approach outperforms the trend test and logistic regression methods for an increased statistical power and reduced false positive inference, which are popularly implemented in the GWAS literature.

## Competing interests

The authors declare that they have no competing interests.

## Authors’ contributions

ZL conceived and designed the study. ZL and MW developed the theoretical analysis. MW, LW, NJ and TJ implemented the simulation and analyzed the PD datasets. ZL and MW wrote the paper. All authors read and approved the final manuscript.

## Supplementary Material

Additional file 1 **Mathematical forms of the coefficients in normal equations (**4**) and (**5**).** Click here for file

Additional file 2 Top two principal components from principal component analysis (PCA) of the stage I dataset. Click here for file

Additional file 3 Association scans from stage II and two-stage combined samples. Click here for file

Additional file 4 Scheme A simulation under dominant and recessive genetic models. Click here for file

Additional file 5 Structured association using logistic regression. Click here for file

Additional file 6 Predicting marker allele frequencies from control samples. Click here for file

Additional file 7 Simulation results from using biased estimates of maker allele frequency. Click here for file
