# Supplementary material for: A robust and efficient statistical method for genetic association studies using case and control samples from multiple cohorts
Source: BMC Genomics. 2013 Feb 8;14:88. doi: 10.1186/1471-2164-14-88 (PMC3626840; doi:10.1186/1471-2164-14-88)
Supplement: Additional file 1 — Mathematical forms of the coefficients in normal equations (4) and (5). [file 1471-2164-14-88-S1.doc]

**Additional file 1 Mathematical forms of the coefficients in normal equations (4) and (5)**

$$\begin{matrix} a_{0}=(2D^{3}+6D^{4}+4D^{5}-2D^{2}p-12D^{3}p-12D^{4}p+6D^{2}p^{2}+12D^{3}p^{2}-4D^{2}p^{3})x_{11} \\ +(2D^{3}+6D^{4}+4D^{5}-2D^{2}p-12D^{3}p-12D^{4}p+6D^{2}p^{2}+12D^{3}p^{2}-4D^{2}p^{3})x_{12} \\ +(2D^{3}+6D^{4}+4D^{5}-2D^{2}p-12D^{3}p-12D^{4}p+6D^{2}p^{2}+12D^{3}p^{2}-4D^{2}p^{3})x_{13} \\ +(D^{3}+3D^{4}+2D^{5}-D^{2}p-6D^{3}p-6D^{4}p+3D^{2}p^{2}+6D^{3}p^{2}-2D^{2}p^{3})x_{21} \\ +(D^{3}+3D^{4}+2D^{5}-D^{2}p-6D^{3}p-6D^{4}p+3D^{2}p^{2}+6D^{3}p^{2}-2D^{2}p^{3})x_{22} \\ +(D^{3}+3D^{4}+2D^{5}-D^{2}p-6D^{3}p-6D^{4}p+3D^{2}p^{2}+6D^{3}p^{2}-2D^{2}p^{3})x_{23} \end{matrix}$$

$$\begin{matrix} a_{1}=(-2D^{2}-14D^{3}-18D^{4}-4D^{5}+2Dp+28D^{2}p+66D^{3}p+32D^{4}p-12Dp^{2}-66D^{2}p^{2} \\ -60D^{3}p^{2}+18Dp^{3}+40D^{2}p^{3}-8Dp^{4})x_{11} \\ +(-D^{2}-11D^{3}-16D^{4}-4D^{5}+Dp+22D^{2}p+60D^{3}p+32D^{4}p-9Dp^{2}-60D^{2}p^{2}-60D^{3}p^{2} \\ +16Dp^{3}+40D^{2}p^{3}-8Dp^{4})x_{12} \\ +(-8D^{3}-14D^{4}-4D^{5}+16D^{2}p+54D^{3}p+32D^{4}p-6Dp^{2}-54D^{2}p^{2}-60D^{3}p^{2}+14Dp^{3} \\ +40D^{2}p^{3}-8Dp^{4})x_{13} \\ +(-D^{2}-8D^{3}-12D^{4}-4D^{5}+Dp+14D^{2}p+36D^{3}p+20D^{4}p-6Dp^{2}-33D^{2}p^{2}-32D^{3}p^{2} \\ +9Dp^{3}+20D^{2}p^{3}-4Dp^{4})x_{21} \\ +(-D^{2}-6D^{3}-10D^{4}-4D^{5}+Dp+12D^{2}p+32D^{3}p+20D^{4}p-5Dp^{2}-30D^{2}p^{2}-32D^{3}p^{2} \\ +8Dp^{3}+20D^{2}p^{3}-4Dp^{4})x_{22} \\ +(-4D^{3}-8D^{4}-4D^{5}+8D^{2}p+28D^{3}p+20D^{4}p-3Dp^{2}-27D^{2}p^{2}-32D^{3}p^{2}+7Dp^{3} \\ +20D^{2}p^{3}-4Dp^{4})x_{23} \\ +(-2D^{3}-6D^{4}-4D^{5}+6D^{3}p+8D^{4}p-4D^{3}p^{2})x_{31} \\ +(-D^{3}-4D^{4}-4D^{5}+4D^{3}p+8D^{4}p-4D^{3}p^{2})x_{32} \\ +(-2D^{4}-4D^{5}+2D^{3}p+8D^{4}p-4D^{3}p^{2})x_{33} \end{matrix}$$

$$\begin{matrix} a_{2}=(8D^{2}+24D^{3}+12D^{4}-14Dp-86D^{2}p-102D^{3}p-20D^{4}p+4p^{2}+72Dp^{2} \\ +186D^{2}p^{2}+88D^{3}p^{2}-12p^{3}-102Dp^{3}-108D^{2}p^{3}+12p^{4}+44Dp^{4}-4p^{5})x_{11} \\ +(4D^{2}+17D^{3}+10D^{4}-7Dp-62D^{2}p-88D^{3}p-20D^{4}p+2p^{2}+51Dp^{2} \\ +162D^{2}p^{2}+88D^{3}p^{2}-8p^{3}-88Dp^{3}-108D^{2}p^{3}+10p^{4}+44Dp^{4}-4p^{5})x_{12} \\ +(10D^{3}+8D^{4}-38D^{2}p-74D^{3}p-20D^{4}p+30Dp^{2}+138D^{2}p^{2}+88D^{3}p^{2} \\ -4p^{3}-74Dp^{3}-108D^{2}p^{3}+8p^{4}+44Dp^{4}-4p^{5})x_{13} \\ +(5D^{2}+18D^{3}+12D^{4}-7Dp-50D^{2}p-72D^{3}p-20D^{4}p+2p^{2}+36Dp^{2} \\ +105D^{2}p^{2}+60D^{3}p^{2}-6p^{3}-51Dp^{3}-60D^{2}p^{3}+6p^{4}+22Dp^{4}-2p^{5})x_{21} \\ +(3D^{2}+12D^{3}+10D^{4}-5Dp-36D^{2}p-60D^{3}p-20D^{4}p+p^{2}+27Dp^{2} \\ +90D^{2}p^{2}+60D^{3}p^{2}-4p^{3}-44Dp^{3}-60D^{2}p^{3}+5p^{4}+22Dp^{4}-2p^{5})x_{22} \\ +(6D^{3}+8D^{4}-20D^{2}p-48D^{3}p-20D^{4}p+15Dp^{2}+75D^{2}p^{2}+60D^{3}p^{2} \\ -2p^{3}-37Dp^{3}-60D^{2}p^{3}+4p^{4}+22Dp^{4}-2p^{5})x_{23} \\ +(2D^{2}+12D^{3}+12D^{4}-14D^{2}p-42D^{3}p-20D^{4}p+24D^{2}p^{2}+32D^{3}p^{2}-12D^{2}p^{3})x_{31} \\ +(D^{2}+7D^{3}+10D^{4}-8D^{2}p-32D^{3}p-20D^{4}p+18D^{2}p^{2}+32D^{3}p^{2}-12D^{2}p^{3})x_{32} \\ +(2D^{3}+8D^{4}-2D^{2}p-22D^{3}p-20D^{4}p+12D^{2}p^{2}+32D^{3}p^{2}-12D^{2}p^{3})x_{33} \end{matrix}$$

$$\begin{matrix} a_{3}=(-10D^{2}-12D^{3}+30Dp+96D^{2}p+48D^{3}p-16p^{2}-144Dp^{2}-198D^{2}p^{2}-40D^{3}p^{2} \\ +48p^{3}+198Dp^{3}+112D^{2}p^{3}-48p^{4}-84Dp^{4}+16p^{5})x_{11} \\ +(-5D^{2}-8D^{3}+15Dp+66D^{2}p+40D^{3}p-8p^{2}-99Dp^{2}-168D^{2}p^{2}-40D^{3}p^{2}+32p^{3} \\ +168Dp^{3}+112D^{2}p^{3}-40p^{4}-84Dp^{4}+16p^{5})x_{12} \\ +(-4D^{3}+36D^{2}p+32D^{3}p-54Dp^{2}-138D^{2}p^{2}-40D^{3}p^{2}+16p^{3}+138Dp^{3}+112D^{2}p^{3} \\ -32p^{4}-84Dp^{4}+16p^{5})x_{13} \\ +(-8D^{2}-12D^{3}+18Dp+72D^{2}p+48D^{3}p-8p^{2}-84Dp^{2}-144D^{2}p^{2}-40D^{3}p^{2}+24p^{3} \\ +114Dp^{3}+80D^{2}p^{3}-24p^{4}-48Dp^{4}+8p^{5})x_{21} \\ +(-4D^{2}-8D^{3}+10Dp+48D^{2}p+40D^{3}p-4p^{2}-58Dp^{2}-120D^{2}p^{2}-40D^{3}p^{2}+16p^{3} \\ +96Dp^{3}+80D^{2}p^{3}-20p^{4}-48Dp^{4}+8p^{5})x_{22} \\ +(-4D^{3}+24D^{2}p+32D^{3}p-30Dp^{2}-96D^{2}p^{2}-40D^{3}p^{2}+8p^{3}+78Dp^{3}+80D^{2}p^{3}-16p^{4} \\ -48Dp^{4}+8p^{5})x_{23} \\ +(-6D^{2}-12D^{3}+6Dp+48D^{2}p+48D^{3}p-24Dp^{2}-90D^{2}p^{2}-40D^{3}p^{2}+30Dp^{3}+48D^{2}p^{3} \\ -12Dp^{4})x_{31} \\ +(-3D^{2}-8D^{3}+3Dp+30D^{2}p+40D^{3}p-15Dp^{2}-72D^{2}p^{2}-40D^{3}p^{2}+24Dp^{3}+48D^{2}p^{3} \\ -12Dp^{4})x_{32} \\ +(-4D^{3}+12D^{2}p+32D^{3}p-6Dp^{2}-54D^{2}p^{2}-40D^{3}p^{2}+18Dp^{3}+48D^{2}p^{3}-12Dp^{4})x_{33} \end{matrix}$$

$$\begin{matrix} a_{4}=(4D^{2}-26Dp-36D^{2}p+24p^{2}+120Dp^{2}+72D^{2}p^{2}-72p^{3}-162Dp^{3}-40D^{2}p^{3} \\ +72p^{4}+68Dp^{4}-24p^{5})x_{11} \\ +(2D^{2}-13Dp-24D^{2}p+12p^{2}+81Dp^{2}+60D^{2}p^{2}-48p^{3}-136Dp^{3}-40D^{2}p^{3}+60p^{4} \\ +68Dp^{4}-24p^{5})x_{12} \\ +(-12D^{2}p+42Dp^{2}+48D^{2}p^{2}-24p^{3}-110Dp^{3}-40D^{2}p^{3}+48p^{4}+68Dp^{4}-24p^{5})x_{13} \\ +(4D^{2}-20Dp-36D^{2}p+14p^{2}+90Dp^{2}+72D^{2}p^{2}-42p^{3}-120Dp^{3}-40D^{2}p^{3}+42p^{4} \\ +50Dp^{4}-14p^{5})x_{21} \\ +(2D^{2}-10Dp-24D^{2}p+7p^{2}+60Dp^{2}+60D^{2}p^{2}-28p^{3}-100Dp^{3}-40D^{2}p^{3}+35p^{4} \\ +50Dp^{4}-14p^{5})x_{22} \\ +(-12D^{2}p+30Dp^{2}+48D^{2}p^{2}-14p^{3}-80Dp^{3}-40D^{2}p^{3}+28p^{4}+50Dp^{4}-14p^{5})x_{23} \\ +(4D^{2}-14Dp-36D^{2}p+4p^{2}+60Dp^{2}+72D^{2}p^{2}-12p^{3}-78Dp^{3}-40D^{2}p^{3}+12p^{4} \\ +32Dp^{4}-4p^{5})x_{31} \\ +(2D^{2}-7Dp-24D^{2}p+2p^{2}+39Dp^{2}+60D^{2}p^{2}-8p^{3}-64Dp^{3}-40D^{2}p^{3}+10p^{4} \\ +32Dp^{4}-4p^{5})x_{32} \\ +(-12D^{2}p+18Dp^{2}+48D^{2}p^{2}-4p^{3}-50Dp^{3}-40D^{2}p^{3}+8p^{4}+32Dp^{4}-4p^{5})x_{33} \end{matrix}$$

$$\begin{matrix} a_{5}=(8Dp-16p^{2}-36Dp^{2}+48p^{3}+48Dp^{3}-48p^{4}-20Dp^{4}+16p^{5})x_{11} \\ +(4Dp-8p^{2}-24Dp^{2}+32p^{3}+40Dp^{3}-40p^{4}-20Dp^{4}+16p^{5})x_{12} \\ +(-12Dp^{2}+16p^{3}+32Dp^{3}-32p^{4}-20Dp^{4}+16p^{5})x_{13} \\ +(8Dp-12p^{2}-36Dp^{2}+36p^{3}+48Dp^{3}-36p^{4}-20Dp^{4}+12p^{5})x_{21} \\ +(4Dp-6p^{2}-24Dp^{2}+24p^{3}+40Dp^{3}-30p^{4}-20Dp^{4}+12p^{5})x_{22} \\ +(-12Dp^{2}+12p^{3}+32Dp^{3}-24p^{4}-20Dp^{4}+12p^{5})x_{23} \\ +(8Dp-8p^{2}-36Dp^{2}+24p^{3}+48Dp^{3}-24p^{4}-20Dp^{4}+8p^{5})x_{31} \\ +(4Dp-4p^{2}-24Dp^{2}+16p^{3}+40Dp^{3}-20p^{4}-20Dp^{4}+8p^{5})x_{32} \\ +(-12Dp^{2}+8p^{3}+32Dp^{3}-16p^{4}-20Dp^{4}+8p^{5})x_{33} \end{matrix}$$

$$\begin{matrix} a_{6}=(4p^{2}-12p^{3}+12p^{4}-4p^{5})x_{11}+(2p^{2}-8p^{3}+10p^{4}-4p^{5})x_{12}+(-4p^{3}+8p^{4}-4p^{5})x_{13} \\ +(4p^{2}-12p^{3}+12p^{4}-4p^{5})x_{21}+(2p^{2}-8p^{3}+10p^{4}-4p^{5})x_{22}+(-4p^{3}+8p^{4}-4p^{5})x_{23} \\ +(4p^{2}-12p^{3}+12p^{4}-4p^{5})x_{31}+(2p^{2}-8p^{3}+10p^{4}-4p^{5})x_{32}+(-4p^{3}+8p^{4}-4p^{5})x_{33} \end{matrix}$$

$$\begin{matrix} b_{0}=(4p^{2}q^{2}-12p^{3}q^{2}+12p^{4}q^{2}-4p^{5}q^{2}-12p^{2}q^{3}+36p^{3}q^{3}-36p^{4}q^{3}+12p^{5}q^{3} \\ +12p^{2}q^{4}-36p^{3}q^{4}+36p^{4}q^{4}-12p^{5}q^{4}-4p^{2}q^{5}+12p^{3}q^{5}-12p^{4}q^{5}+4p^{5}q^{5})x_{11} \\ +(2p^{2}q^{2}-8p^{3}q^{2}+10p^{4}q^{2}-4p^{5}q^{2}-6p^{2}q^{3}+24p^{3}q^{3}-30p^{4}q^{3}+12p^{5}q^{3}+6p^{2}q^{4} \\ -24p^{3}q^{4}+30p^{4}q^{4}-12p^{5}q^{4}-2p^{2}q^{5}+8p^{3}q^{5}-10p^{4}q^{5}+4p^{5}q^{5})x_{12} \\ +(-4p^{3}q^{2}+8p^{4}q^{2}-4p^{5}q^{2}+12p^{3}q^{3}-24p^{4}q^{3}+12p^{5}q^{3}-12p^{3}q^{4}+24p^{4}q^{4}-12p^{5}q^{4} \\ +4p^{3}q^{5}-8p^{4}q^{5}+4p^{5}q^{5})x_{13} \\ +(2p^{2}q^{2}-6p^{3}q^{2}+6p^{4}q^{2}-2p^{5}q^{2}-8p^{2}q^{3}+24p^{3}q^{3}-24p^{4}q^{3}+8p^{5}q^{3}+10p^{2}q^{4}-30p^{3}q^{4} \\ +30p^{4}q^{4}-10p^{5}q^{4}-4p^{2}q^{5}+12p^{3}q^{5}-12p^{4}q^{5}+4p^{5}q^{5})x_{21} \\ +(p^{2}q^{2}-4p^{3}q^{2}+5p^{4}q^{2}-2p^{5}q^{2}-4p^{2}q^{3}+16p^{3}q^{3}-20p^{4}q^{3}+8p^{5}q^{3}+5p^{2}q^{4}-20p^{3}q^{4} \\ +25p^{4}q^{4}-10p^{5}q^{4}-2p^{2}q^{5}+8p^{3}q^{5}-10p^{4}q^{5}+4p^{5}q^{5})x_{22} \\ +(-2p^{3}q^{2}+4p^{4}q^{2}-2p^{5}q^{2}+8p^{3}q^{3}-16p^{4}q^{3}+8p^{5}q^{3}-10p^{3}q^{4}+20p^{4}q^{4}-10p^{5}q^{4} \\ +4p^{3}q^{5}-8p^{4}q^{5}+4p^{5}q^{5})x_{23} \\ +(-4p^{2}q^{3}+12p^{3}q^{3}-12p^{4}q^{3}+4p^{5}q^{3}+8p^{2}q^{4}-24p^{3}q^{4}+24p^{4}q^{4}-8p^{5}q^{4}-4p^{2}q^{5} \\ +12p^{3}q^{5}-12p^{4}q^{5}+4p^{5}q^{5})x_{31} \\ +(-2p^{2}q^{3}+8p^{3}q^{3}-10p^{4}q^{3}+4p^{5}q^{3}+4p^{2}q^{4}-16p^{3}q^{4}+20p^{4}q^{4}-8p^{5}q^{4}-2p^{2}q^{5}+8p^{3}q^{5} \\ -10p^{4}q^{5}+4p^{5}q^{5})x_{32} \\ +(4p^{3}q^{3}-8p^{4}q^{3}+4p^{5}q^{3}-8p^{3}q^{4}+16p^{4}q^{4}-8p^{5}q^{4}+4p^{3}q^{5}-8p^{4}q^{5}+4p^{5}q^{5})x_{33} \end{matrix}$$

$$\begin{matrix} b_{1}=(2pq-12p^{2}q+18p^{3}q-8p^{4}q-12pq^{2}+60p^{2}q^{2}-84p^{3}q^{2}+36p^{4}q^{2}+18pq^{3}-84p^{2}q^{3} \\ +114p^{3}q^{3}-48p^{4}q^{3}-8pq^{4}+36p^{2}q^{4}-48p^{3}q^{4}+20p^{4}q^{4})x_{11} \\ +(pq-9p^{2}q+16p^{3}q-8p^{4}q-6pq^{2}+42p^{2}q^{2}-72p^{3}q^{2}+36p^{4}q^{2}+9pq^{3}-57p^{2}q^{3}+96p^{3}q^{3} \\ -48p^{4}q^{3}-4pq^{4}+24p^{2}q^{4}-40p^{3}q^{4}+20p^{4}q^{4})x_{12} \\ +(-6p^{2}q+14p^{3}q-8p^{4}q+24p^{2}q^{2}-60p^{3}q^{2}+36p^{4}q^{2}-30p^{2}q^{3}+78p^{3}q^{3}-48p^{4}q^{3}+12p^{2}q^{4} \\ -32p^{3}q^{4}+20p^{4}q^{4})x_{13} \\ +(pq-6p^{2}q+9p^{3}q-4p^{4}q-9pq^{2}+42p^{2}q^{2}-57p^{3}q^{2}+24p^{4}q^{2}+16pq^{3}-72p^{2}q^{3}+96p^{3}q^{3} \\ -40p^{4}q^{3}-8pq^{4}+36p^{2}q^{4}-48p^{3}q^{4}+20p^{4}q^{4})x_{21} \\ +(pq-5p^{2}q+8p^{3}q-4p^{4}q-5pq^{2}+29p^{2}q^{2}-48p^{3}q^{2}+24p^{4}q^{2}+8pq^{3}-48p^{2}q^{3}+80p^{3}q^{3} \\ -40p^{4}q^{3}-4pq^{4}+24p^{2}q^{4}-40p^{3}q^{4}+20p^{4}q^{4})x_{22} \\ +(-3p^{2}q+7p^{3}q-4p^{4}q+15p^{2}q^{2}-39p^{3}q^{2}+24p^{4}q^{2}-24p^{2}q^{3}+64p^{3}q^{3}-40p^{4}q^{3}+12p^{2}q^{4} \\ -32p^{3}q^{4}+20p^{4}q^{4})x_{23} \\ +(-6pq^{2}+24p^{2}q^{2}-30p^{3}q^{2}+12p^{4}q^{2}+14pq^{3}-60p^{2}q^{3}+78p^{3}q^{3}-32p^{4}q^{3}-8pq^{4}+36p^{2}q^{4} \\ -48p^{3}q^{4}+20p^{4}q^{4})x_{31} \\ +(-3pq^{2}+15p^{2}q^{2}-24p^{3}q^{2}+12p^{4}q^{2}+7pq^{3}-39p^{2}q^{3}+64p^{3}q^{3}-32p^{4}q^{3}-4pq^{4}+24p^{2}q^{4} \\ -40p^{3}q^{4}+20p^{4}q^{4})x_{32} \\ +(6p^{2}q^{2}-18p^{3}q^{2}+12p^{4}q^{2}-18p^{2}q^{3}+50p^{3}q^{3}-32p^{4}q^{3}+12p^{2}q^{4}-32p^{3}q^{4}+20p^{4}q^{4})x_{33} \end{matrix}$$

$$\begin{matrix} b_{2}=(-2p+6p^{2}-4p^{3}-2q+26pq-60p^{2}q+36p^{3}q+6q^{2}-60pq^{2}+126p^{2}q^{2}-72p^{3}q^{2} \\ -4q^{3}+36pq^{3}-72p^{2}q^{3}+40p^{3}q^{3})x_{11} \\ +(-2p+6p^{2}-4p^{3}-q+20pq-54p^{2}q+36p^{3}q+3q^{2}-42pq^{2}+108p^{2}q^{2}-72p^{3}q^{2}-2q^{3} \\ +24pq^{3}-60p^{2}q^{3}+40p^{3}q^{3})x_{12} \\ +(-2p+6p^{2}-4p^{3}+14pq-48p^{2}q+36p^{3}q-24pq^{2}+90p^{2}q^{2}-72p^{3}q^{2}+12pq^{3}-48p^{2}q^{3} \\ +40p^{3}q^{3})x_{13} \\ +(-p+3p^{2}-2p^{3}-2q+20pq-42p^{2}q+24p^{3}q+6q^{2}-54pq^{2}+108p^{2}q^{2}-60p^{3}q^{2}-4q^{3} \\ +36pq^{3}-72p^{2}q^{3}+40p^{3}q^{3})x_{21} \\ +(-p+3p^{2}-2p^{3}-q+14pq-36p^{2}q+24p^{3}q+3q^{2}-36pq^{2}+90p^{2}q^{2}-60p^{3}q^{2}-2q^{3} \\ +24pq^{3}-60p^{2}q^{3}+40p^{3}q^{3})x_{22} \\ +(-p+3p^{2}-2p^{3}+8pq-30p^{2}q+24p^{3}q-18pq^{2}+72p^{2}q^{2}-60p^{3}q^{2}+12pq^{3}-48p^{2}q^{3} \\ +40p^{3}q^{3})x_{23} \\ +(-2q+14pq-24p^{2}q+12p^{3}q+6q^{2}-48pq^{2}+90p^{2}q^{2}-48p^{3}q^{2}-4q^{3}+36pq^{3}-72p^{2}q^{3} \\ +40p^{3}q^{3})x_{31} \\ +(-q+8pq-18p^{2}q+12p^{3}q+3q^{2}-30pq^{2}+72p^{2}q^{2}-48p^{3}q^{2}-2q^{3}+24pq^{3}-60p^{2}q^{3} \\ +40p^{3}q^{3})x_{32} \\ +(2pq-12p^{2}q+12p^{3}q-12pq^{2}+54p^{2}q^{2}-48p^{3}q^{2}+12pq^{3}-48p^{2}q^{3}+40p^{3}q^{3})x_{33} \end{matrix}$$

$$\begin{matrix} b_{3}=(2-12p+12p^{2}-12q+54pq-48p^{2}q+12q^{2}-48pq^{2}+40p^{2}q^{2})x_{11} \\ +(2-12p+12p^{2}-9q+48pq-48p^{2}q+8q^{2}-40pq^{2}+40p^{2}q^{2})x_{12} \\ +(2-12p+12p^{2}-6q+42pq-48p^{2}q+4q^{2}-32pq^{2}+40p^{2}q^{2})x_{13} \\ +(2-9p+8p^{2}-12q+48pq-40p^{2}q+12q^{2}-48pq^{2}+40p^{2}q^{2})x_{21} \\ +(1-8p+8p^{2}-8q+40pq-40p^{2}q+8q^{2}-40pq^{2}+40p^{2}q^{2})x_{22} \\ +(1-7p+8p^{2}-4q+32pq-40p^{2}q+4q^{2}-32pq^{2}+40p^{2}q^{2})x_{23} \\ +(2-6p+4p^{2}-12q+42pq-32p^{2}q+12q^{2}-48pq^{2}+40p^{2}q^{2})x_{31} \\ +(1-4p+4p^{2}-7q+32pq-32p^{2}q+8q^{2}-40pq^{2}+40p^{2}q^{2})x_{32} \\ +(-2p+4p^{2}-2q+22pq-32p^{2}q+4q^{2}-32pq^{2}+40p^{2}q^{2})x_{33} \end{matrix}$$

$$\begin{matrix} b_{4}=(6-12p-12q+20pq)x_{11}+(6-12p-10q+20pq)x_{12}+(6-12p-8q+20pq)x_{13} \\ +(6-10p-12q+20pq)x_{21}+(5-10p-10q+20pq)x_{22}+(4-10p-8q+20pq)x_{23} \\ +(6-8p-12q+20pq)x_{31}+(4-8p-10q+20pq)x_{32}+(2-8p-8q+20pq)x_{33} \end{matrix}$$

$$b_{5}=4n$$

where $x_{ij}=n_{1j}w_{ij}\text{+}n_{2j}v_{ij}$.
