# Supplementary material for: A robust and efficient statistical method for genetic association studies using case and control samples from multiple cohorts
Source: BMC Genomics. 2013 Feb 8;14:88. doi: 10.1186/1471-2164-14-88 (PMC3626840; doi:10.1186/1471-2164-14-88)
Supplement: Additional file 2 — Top two principal components from principal component analysis (PCA) of the stage I dataset. [file 1471-2164-14-88-S2.doc]

**
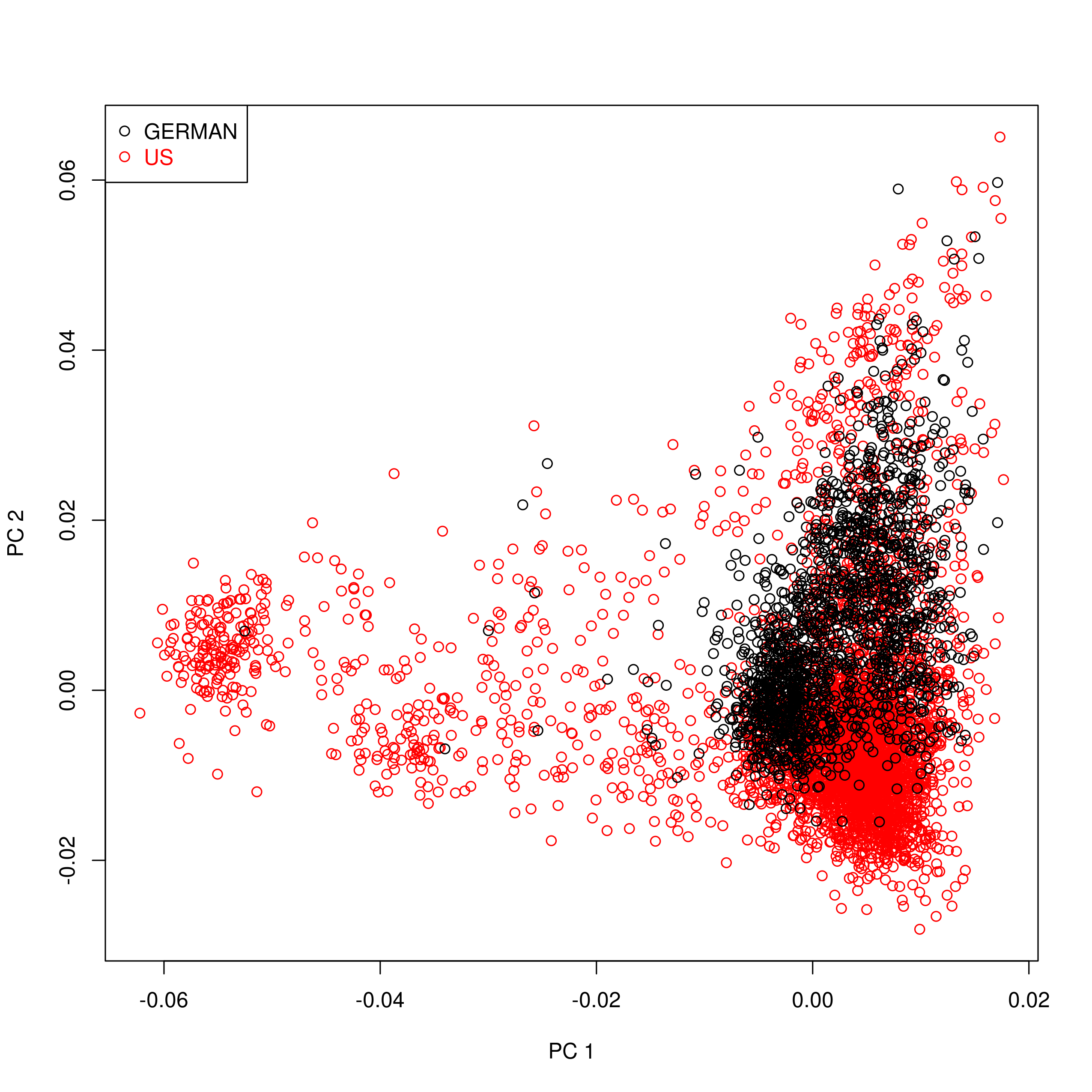
**

**Additional file 2** **Top two principal components from principal component analysis (PCA) of the stage I dataset**
