# Supplementary material for: A robust and efficient statistical method for genetic association studies using case and control samples from multiple cohorts
Source: BMC Genomics. 2013 Feb 8;14:88. doi: 10.1186/1471-2164-14-88 (PMC3626840; doi:10.1186/1471-2164-14-88)
Supplement: Additional file 3 — Association scans from stage II and two-stage combined samples. [file 1471-2164-14-88-S3.doc]

**Additional file 3 Association scans from stage II and two-stage combined samples**

a.

| Locus | SNP | Position (bp) | *P* value | | |
| --- | --- | --- | --- | --- | --- |
|  |  |  | Method 1 | Method 2 | Method 3 |
| 4q21 | rs356229 | 90825620 | 3.5×10^-7^ | 6.3×10^-4^ | 2.6×10^-6^ |
|  | rs11931074 | 90858538 | 2.1×10^-4^ | 4.1×10^-7^ | 3.7×10^-8^ |
|  | rs3857059 | 90894261 | 1.9×10^-4^ | 3.4×10^-7^ | 3.4×10^-8^ |
|  | rs2736990 | 90897564 | 1.9×10^-12^ | 2.1×10^-7^ | 5.5×10^-9^ |
|  | rs3775439 | 90928764 | 2.1×10^-5^ | 7.0×10^-3^ | 1.5×10^-3^ |
|  | rs894278 | 90953558 | 2.1×10^-3^ | 2.6×10^-4^ | 5.9×10^-5^ |
| 5p15.2 | rs26286 | 14219402 | 1.7×10^-5^ | 0.025 | 0.044 |
| 7p21.3 | rs2681051 | 11615690 | 1.5×10^-5^ | 0.60 | 0.31 |
| 11q12-q13.1 | rs1005511 | 57123232 | 6.8×10^-7^ | 0.14 | 0.25 |
| 12q12 | rs11564162 | 38729159 | 2.2×10^-5^ | 0.019 | 0.095 |
| 14q22.2 | rs2878172 | 54443420 | 1.3×10^-7^ | 0.06 | 0.12 |
| 17q21 | rs11012 | 40869224 | 3.0×10^-12^ | 4.0×10^-6^ | 2.1×10^-5^ |
|  | rs393152 | 41074926 | 7.9×10^-20^ | 7.2×10^-11^ | 1.7×10^-9^ |
|  | rs417968 | 41084159 | 1.3×10^-12^ | 2.2×10^-7^ | 4.5×10^-7^ |
|  | rs7215239 | 41123556 | 8.8×10^-15^ | 9.4×10^-8^ | 4.8×10^-7^ |
|  | rs12373139 | 41279910 | 2.8×10^-15^ | 1.9×10^-8^ | 1.2×10^-7^ |
|  | rs17690703 | 41281077 | 1.5×10^-18^ | 2.9×10^-9^ | 2.3×10^-8^ |
|  | rs17563986 | 41347100 | 1.3×10^-16^ | 4.8×10^-9^ | 3.2×10^-8^ |
|  | rs1981997 | 41412603 | 6.4×10^-16^ | 1.1×10^-8^ | 9.1×10^-8^ |
|  | rs8070723 | 41436901 | 9.0×10^-16^ | 1.1×10^-8^ | 9.2×10^-8^ |
|  | rs7225002 | 41544850 | 7.5×10^-9^ | 8.5×10^-5^ | 5.8×10^-4^ |
|  | rs2532274 | 41602941 | 7.4×10^-10^ | 3.6×10^-6^ | 1.6×10^-5^ |
|  | rs2532269 | 41605885 | 8.8×10^-15^ | 8.9×10^-8^ | 6.2×10^-7^ |
|  | rs2668692 | 41648797 | 4.2×10^-16^ | 7.7×10^-9^ | 5.3×10^-8^ |
|  | rs183211 | 42143493 | 2.0×10^-10^ | 6.3×10^-7^ | 3.9×10^-6^ |
|  | rs169201 | 42145386 | 9.0×10^-16^ | 1.0×10^-8^ | 8.8×10^-8^ |
|  | rs7224296 | 42155230 | 9.4×10^-9^ | 1.1×10^-5^ | 3.3×10^-5^ |
|  | rs199533 | 42184098 | 2.3×10^-16^ | 7.4×10^-9^ | 8.0×10^-8^ |
| 21q22.3 | rs681210 | 43603771 | 1.1×10^-5^ | 0.60 | 0.43 |

b.

| Locus | SNP | Position (bp) | *P* value | | |
| --- | --- | --- | --- | --- | --- |
|  |  |  | Method 1 | Method 2 | Method 3 |
| 1p21.1 | rs12172730 | 104913058 | 1.6×10^-4^ | 2.2×10^-4^ | 1.1×10^-4^ |
| 2p25.3 | rs6542651 | 3737705 | 4.0×10^-4^ | 3.6×10^-5^ | 1.4×10^-5^ |
| 2p24.2 | rs2042079 | 16969333 | 1.3×10^-6^ | 1.4×10^-5^ | 8.2×10^-6^ |
| 2p22.3 | rs935920 | 35985642 | 3.2×10^-7^ | 2.3×10^-5^ | 2.5×10^-5^ |
| 2p22.3 | rs2949065 | 35986447 | 5.7×10^-7^ | 3.8×10^-5^ | 4.5×10^-5^ |
| 2p21 | rs935378 | 46962655 | 3.8×10^-6^ | 6.5×10^-4^ | 4.7×10^-4^ |
| 2q36.3 | rs1035833 | 230417442 | 1.2×10^-4^ | 4.2×10^-2^ | 2.7×10^-2^ |
| 3p25.1 | rs7651825 | 14787122 | 2.2×10^-6^ | 5.6×10^-5^ | 5.2×10^-5^ |
| 3q23 | rs6440096 | 143814050 | 3.3×10^-6^ | 2.9×10^-4^ | 4.3×10^-4^ |
|  | rs6800573 | 143828507 | 1.4×10^-5^ | 3.2×10^-4^ | 5.7×10^-4^ |
| 4p15.2 | rs1453815 | 24566751 | 9.0×10^-5^ | 1.0×10^-3^ | 7.1×10^-4^ |
|  | rs4697508 | 24576450 | 2.9×10^-5^ | 7.4×10^-4^ | 5.8×10^-4^ |
| 4q21.1 | rs7666265 | 77395305 | 1.4×10^-4^ | 3.1×10^-4^ | 5.4×10^-4^ |
|  | rs6851219 | 77398854 | 2.8×10^-6^ | 2.9×10^-5^ | 3.6×10^-5^ |
|  | rs6812193 | 77418010 | 8.5×10^-8^ | 6.7×10^-7^ | 5.9×10^-7^ |
| 4q21 | rs1430961 | 90771943 | 5.6×10^-6^ | 3.6×10^-5^ | 4.4×10^-6^ |
|  | rs12644119 | 90822442 | 1.2×10^-9^ | 3.8×10^-7^ | 2.3×10^-8^ |
|  | rs356229 | 90825620 | 6.4×10^-11^ | 5.5×10^-7^ | 2.3×10^-9^ |
|  | rs11931074 | 90858538 | 7.0×10^-19^ | 2.3×10^-14^ | 2.6×10^-15^ |
|  | rs3857059 | 90894261 | 2.4×10^-19^ | 1.6×10^-14^ | 1.9×10^-15^ |
|  | rs2736990 | 90897564 | 5.7×10^-23^ | 6.1×10^-15^ | 3.3×10^-16^ |
|  | rs3775439 | 90928764 | 2.8×10^-11^ | 2.2×10^-8^ | 2.2×10^-8^ |
|  | rs894278 | 90953558 | 7.4×10^-11^ | 1.4×10^-8^ | 2.9×10^-9^ |
| 4q22 | rs6532197 | 91016324 | 2.1×10^-8^ | 2.1×10^-7^ | 5.7×10^-8^ |
| 5p15.2 | rs26286 | 14219402 | 5.9×10^-9^ | 7.2×10^-5^ | 4.7×10^-5^ |
| 5q11.2-q13.3 | rs3792738 | 76283540 | 2.8×10^-6^ | 1.7×10^-5^ | 2.5×10^-5^ |
| 5q23.3 | rs264122 | 129675680 | 5.8×10^-5^ | 1.6×10^-3^ | 6.6×10^-3^ |
| 6q22 | rs6903627 | 120169406 | 1.0×10^-4^ | 3.0×10^-2^ | 4.9×10^-2^ |
| 6q23.3 | rs996243 | 137895153 | 5.4×10^-6^ | 1.5×10^-4^ | 9.0×10^-4^ |
| 7p21.3 | rs2681051 | 11615690 | 4.1×10^-5^ | 4.0×10^-2^ | 1.3×10^-1^ |
| 7p12.3 | rs2708909 | 48018204 | 1.4×10^-5^ | 3.6×10^-3^ | 2.6×10^-3^ |
|  | rs2708851 | 48052327 | 2.8×10^-5^ | 4.4×10^-3^ | 3.8×10^-3^ |
| 8p21.2 | rs925030 | 25298518 | 1.3×10^-4^ | 3.7×10^-4^ | 3.4×10^-4^ |
| 9p24.3 | rs4742236 | 676753 | 7.6×10^-5^ | 3.8×10^-4^ | 3.3×10^-4^ |
|  | rs9299039 | 680460 | 7.9×10^-5^ | 1.2×10^-3^ | 6.6×10^-4^ |
| 9p21.2 | rs4534200 | 25642508 | 8.6×10^-5^ | 1.5×10^-3^ | 2.0×10^-3^ |
|  | rs700802 | 78424532 | 2.5×10^-5^ | 2.6×10^-3^ | 2.9×10^-3^ |
| 9q21.31 | rs7024926 | 82766092 | 1.0×10^-5^ | 1.1×10^-3^ | 4.6×10^-4^ |
|  | rs2378554 | 82805138 | 2.3×10^-5^ | 4.2×10^-4^ | 5.0×10^-4^ |
|  | rs9918939 | 82836491 | 3.2×10^-5^ | 4.2×10^-4^ | 4.7×10^-4^ |
| 9q34.11 | rs2240914 | 131938127 | 9.1×10^-5^ | 1.1×10^-2^ | 1.5×10^-2^ |
| 9q34.2 | rs11185726 | 136240925 | 6.0×10^-7^ | 3.3×10^-4^ | 1.4×10^-3^ |
| 10p13 | rs7077361 | 15601549 | 4.7×10^-9^ | 3.0×10^-5^ | 7.3×10^-6^ |
| 10p12.1 | rs11595185 | 25231376 | 1.0×10^-7^ | 2.3×10^-5^ | 2.5×10^-5^ |
| 10p11.21 | rs11591754 | 35247159 | 3.2×10^-6^ | 9.4×10^-5^ | 2.7×10^-4^ |
| 10q22.1 | rs2491015 | 70436819 | 8.2×10^-7^ | 1.0×10^-4^ | 2.1×10^-4^ |
| 10q24.32 | rs999867 | 104494554 | 6.6×10^-8^ | 2.6×10^-5^ | 1.8×10^-6^ |
|  | rs17115100 | 104581383 | 1.4×10^-8^ | 3.6×10^-6^ | 1.4×10^-7^ |
|  | rs3824754 | 104604340 | 4.6×10^-7^ | 5.1×10^-5^ | 3.6×10^-6^ |
|  | rs4409766 | 104606653 | 2.6×10^-7^ | 2.2×10^-5^ | 1.3×10^-6^ |
|  | rs11191425 | 104615960 | 1.2×10^-7^ | 1.5×10^-5^ | 5.4×10^-7^ |
|  | rs12411886 | 104675289 | 9.0×10^-7^ | 3.7×10^-5^ | 1.8×10^-6^ |
|  | rs12413409 | 104709086 | 8.6×10^-7^ | 3.6×10^-5^ | 1.6×10^-6^ |
| 10q26 | rs12777747 | 123989646 | 1.0×10^-5^ | 2.7×10^-4^ | 2.5×10^-4^ |
| 11q12-q13.1 | rs1005511 | 57123232 | 1.3×10^-9^ | 5.3×10^-4^ | 5.9×10^-4^ |
| 12p12.1 | rs699038 | 25050907 | 1.5×10^-5^ | 2.8×10^-4^ | 2.8×10^-4^ |
| 12q12 | rs11564162 | 38729159 | 3.9×10^-9^ | 2.1×10^-5^ | 1.3×10^-4^ |
|  | rs1491923 | 38877384 | 2.5×10^-5^ | 5.3×10^-5^ | 1.5×10^-5^ |
| 12q13.11 | rs1793949 | 46657862 | 1.8×10^-6^ | 3.0×10^-4^ | 2.1×10^-4^ |
| 12q13.2 | rs2710697 | 53684257 | 3.6×10^-6^ | 1.3×10^-3^ | 4.8×10^-3^ |
| 13q14.11 | rs9525776 | 42928966 | 6.7×10^-6^ | 5.4×10^-2^ | 4.3×10^-2^ |
| 13q22.2 | rs9530494 | 75434275 | 2.1×10^-5^ | 4.6×10^-4^ | 3.5×10^-4^ |
| 14q22-q23 | rs2150279 | 53343338 | 9.0×10^-5^ | 1.2×10^-3^ | 6.7×10^-4^ |
|  | rs12431733 | 53360580 | 4.5×10^-8^ | 3.5×10^-6^ | 2.3×10^-6^ |
| 14q22.2 | rs2878172 | 54443420 | 1.2×10^-9^ | 2.8×10^-4^ | 6.4×10^-4^ |
| 15q22 | rs1481088 | 71629314 | 1.4×10^-5^ | 5.4×10^-5^ | 4.7×10^-5^ |
|  | rs922687 | 71635861 | 1.3×10^-6^ | 2.1×10^-5^ | 1.4×10^-5^ |
| 17p13.3 | rs4247113 | 228978 | 4.2×10^-5^ | 2.3×10^-4^ | 9.8×10^-4^ |
| 17q21 | rs11012 | 40869224 | 1.1×10^-16^ | 9.7×10^-10^ | 1.7×10^-10^ |
|  | rs393152 | 41074926 | 5.5×10^-27^ | 5.0×10^-15^ | 4.4×10^-16^ |
|  | rs417968 | 41084159 | 6.2×10^-17^ | 4.7×10^-10^ | 7.0×10^-11^ |
|  | rs7215239 | 41123556 | 1.5×10^-19^ | 1.4×10^-10^ | 3.0×10^-11^ |
|  | rs1526123 | 41139123 | 5.9×10^-8^ | 1.4×10^-6^ | 1.3×10^-7^ |
|  | rs12373139 | 41279910 | 9.7×10^-23^ | 4.7×10^-13^ | 1.5×10^-13^ |
|  | rs17690703 | 41281077 | 3.4×10^-22^ | 6.9×10^-12^ | 1.4×10^-12^ |
|  | rs17563986 | 41347100 | 3.3×10^-24^ | 1.0×10^-13^ | 2.4×10^-14^ |
|  | rs1981997 | 41412603 | 1.2×10^-23^ | 2.0×10^-13^ | 5.3×10^-14^ |
|  | rs8070723 | 41436901 | 4.5×10^-23^ | 3.6×10^-13^ | 1.0×10^-13^ |
|  | rs7225002 | 41544850 | 3.1×10^-13^ | 7.4×10^-8^ | 1.0×10^-7^ |
|  | rs2532274 | 41602941 | 3.8×10^-16^ | 1.9×10^-10^ | 1.2×10^-10^ |
|  | rs2532269 | 41605885 | 3.6×10^-22^ | 3.1×10^-12^ | 9.5×10^-13^ |
|  | rs2668692 | 41648797 | 4.7×10^-23^ | 3.7×10^-13^ | 1.2×10^-13^ |
|  | rs183211 | 42143493 | 4.2×10^-16^ | 3.4×10^-10^ | 1.4×10^-10^ |
|  | rs169201 | 42145386 | 6.2×10^-24^ | 1.1×10^-13^ | 5.9×10^-14^ |
|  | rs7224296 | 42155230 | 2.8×10^-13^ | 4.5×10^-8^ | 1.8×10^-8^ |
|  | rs199533 | 42184098 | 7.7×10^-25^ | 3.9×10^-14^ | 2.5×10^-14^ |
| 20p12.1 | rs1223271 | 13244912 | 6.0×10^-7^ | 1.3×10^-5^ | 6.8×10^-6^ |
| 21q22.3 | rs681210 | 43603771 | 7.4×10^-6^ | 3.9×10^-3^ | 2.2×10^-3^ |
|  | rs595046 | 43626445 | 1.3×10^-4^ | 1.5×10^-2^ | 2.4×10^-2^ |
| 22q13.32 | rs4823506 | 46643740 | 1.0×10^-4^ | 4.9×10^-3^ | 6.5×10^-3^ |

SNPs surpassed Bonferroni genome-wide threshold (1.5×10^-4^) in the analysis of (a) stage II and (b) two-stage combined Parkinson’s disease case and control samples.
