# Supplementary material for: A robust and efficient statistical method for genetic association studies using case and control samples from multiple cohorts
Source: BMC Genomics. 2013 Feb 8;14:88. doi: 10.1186/1471-2164-14-88 (PMC3626840; doi:10.1186/1471-2164-14-88)
Supplement: Additional file 4 — Scheme A simulation under dominant and recessive genetic models. [file 1471-2164-14-88-S4.doc]

**Additional file 4 Scheme A simulation under dominant and recessive genetic models**

a.

| Pop. |  | Method 1 | | | |  | | Method 3 | |
| --- | --- | --- | --- | --- | --- | --- | --- | --- | --- |
|  | $\hat{q}\pm s.d.$ | | $\hat{D}\pm s.d.$ | $\chi_{[2]}^{2}\pm s.d.$ | *ρ* (%) | | $\chi_{[1]}^{2}\pm s.d.$ | | *ρ* (%) |
| 1 | - | | 0.001±0.010 | 1.7±2.1 | 0 | | 1.0±1.4 | | 0 |
| 2 | - | | 0.001±0.009 | 1.8±2.0 | 0.1 | | 1.0±1.3 | | 0 |
| 3 | - | | 0.001±0.009 | 1.8±1.9 | 0 | | 1.0±1.4 | | 0 |
| 4 | 0.51±0.06 | | 0.147±0.017 | 225.3±31.8 | 100 | | 115.9±17.2 | | 100 |
| 5 | 0.51±0.09 | | 0.097±0.018 | 93.4±21.2 | 100 | | 47.5±12.8 | | 99.9 |
| 6 | 0.54±0.18 | | 0.044±0.018 | 23.3±10.1 | 58.7 | | 11.5±6.4 | | 20.5 |
| 7 | 0.81±0.13 | | 0.044±0.031 | 148.2±20.0 | 100 | | 80.1±15.7 | | 100 |
| 8 | 0.73±0.16 | | 0.045±0.026 | 69.8±15.9 | 100 | | 35.4±11.2 | | 96.7 |
| 9 | 0.30±0.06 | | -0.069±0.011 | 48.7±15.1 | 99.0 | | 12.8±6.0 | | 23.9 |
| 10 | 0.31±0.10 | | -0.048±0.012 | 24.9±10.4 | 65.1 | | 7.5±4.8 | | 4.9 |

b.

| Pop. |  | Method 1 | | | |  | | Method 3 | |
| --- | --- | --- | --- | --- | --- | --- | --- | --- | --- |
|  | $\hat{q}\pm s.d.$ | | $\hat{D}\pm s.d.$ | $\chi_{[2]}^{2}\pm s.d.$ | *ρ* (%) | | $\chi_{[1]}^{2}\pm s.d.$ | | *ρ* (%) |
| 1 | - | | 0.001±0.010 | 1.7±2.0 | 0 | | 1.0±1.4 | | 0 |
| 2 | - | | 0.001±0.009 | 1.7±2.0 | 0 | | 1.0±1.3 | | 0 |
| 3 | - | | 0.001±0.009 | 1.8±2.0 | 0 | | 1.0±1.4 | | 0 |
| 4 | 0.49±0.06 | | 0.147±0.018 | 224.9±31.6 | 100 | | 115.7±17.2 | | 100 |
| 5 | 0.44±0.16 | | 0.089±0.032 | 91.8±23.9 | 100 | | 47.6±12.8 | | 100 |
| 6 | 0.45±0.18 | | 0.044±0.018 | 23.4±10.5 | 58.7 | | 11.3±6.4 | | 19.2 |
| 7 | 0.70±0.06 | | 0.069±0.013 | 55.9±16.7 | 99.3 | | 23.0±7.0 | | 82.5 |
| 8 | 0.69±0.09 | | 0.048±0.013 | 27.2±12.3 | 68.9 | | 11.5±5.9 | | 19.8 |
| 9 | 0.27±0.14 | | -0.061±0.032 | 111.9±25.3 | 99.9 | | 39.8±12.1 | | 99.0 |
| 10 | 0.28±0.19 | | -0.043±0.027 | 57.5±18.4 | 97.8 | | 22.1±8.8 | | 72.5 |

Results from scheme A simulation under (a) dominant and (b) recessive genetic model. Population number and notations are the same as in Table 3.
