# Supplementary material for: A robust and efficient statistical method for genetic association studies using case and control samples from multiple cohorts
Source: BMC Genomics. 2013 Feb 8;14:88. doi: 10.1186/1471-2164-14-88 (PMC3626840; doi:10.1186/1471-2164-14-88)
Supplement: Additional file 5 — Structured association using logistic regression. [file 1471-2164-14-88-S5.doc]

**Additional file 5 Structured association using logistic regression**

Let us consider a case and control sample consisting of *k* distinct case and control cohorts. The number of individuals from the *i*th corhort is denoted by *n*(*^i^*), in which $n_{i}^{case}$are cases and $n_{i}^{control}$are controls. Suppose the sample has been genotyped at a bi-allelic SNP. Let *Z_ij_* ∈ (0, 1, 2) denote the genotype for the given coding of the two alleles at the SNP and *Y_ij_* be the binary disease phenotype (*Y_ij_* = 1 for cases and *Y_ij_* = 0 for controls) for individual *j* from the *i*th cohort. The widely used logistic regression approach for testing for the association between disease phenotype and marker genotype can be formulated as

$L(\theta)=P(Y|Z,\alpha,\gamma)=\prod_{i=1}^{k} \prod_{j=1}^{n^{(i)}} f_{ij}^{Y_{ij}}(1-f_{ij})^{1-Y_{ij}}$ (S1)

where

$\theta=(\alpha,\gamma)$, $\eta_{ij}(\theta)=\log(\frac{f_{ij}}{1-f_{ij}})=\alpha_{j}+\gamma Z_{ij}$ and $f_{ij}=\frac{e^{\eta_{ij}(\theta)}}{1+e^{\eta_{ij}(\theta)}}$

in which, $\alpha=(\alpha_{1},\alpha_{2},...,\alpha_{k})$ are the baseline log-odds of disease for the reference genotypes (genotypes 0 here) in the *k* cohorts, *γ* specifies the increase in log-odds caused by each copy of the allele coded 1 and *f_ij_* is the probability that individual *j* from the *i*th cohort develops the disease. To test for the association we can test the hypotheses

$$H_{0}:\gamma=0\text{ vs }H_{1}:\gamma\neq0$$

through either the likelihood ratio (LR) test or the Score Test. The Score Test utilises the first and second partial derivatives of the logarithm of the likelihood function evaluated with parameters estimated under the null hypothesis *H*_0_: *γ* = 0. For the present logistic regression model, the first partial derivative of the logarithm of the likelihood function with respect to parameters *θ* is

$\frac{dl(\theta)}{d\theta}=\sum_{i=1}^{k} \sum_{j=1}^{n^{(i)}} (Y_{ij}-f_{ij})\frac{d\eta_{ij}(\theta)}{d\theta}$ (S2)

, from which, we have

$\frac{dl(\theta)}{d\alpha_{i}}=\sum_{j=1}^{n^{(i)}} (Y_{ij}-f_{ij})$ and $\frac{dl(\theta)}{d\gamma}=\sum_{i=1}^{k} \sum_{j=1}^{n^{(i)}} (Y_{ij}-f_{ij})Z_{ij}$.

The second partial derivatives of the logarithmic likelihood function with respect to parameters *θ* are

$\frac{dl^{2}(\theta)}{d\alpha_{i}d\alpha_{l}}=\left\{\begin{matrix} -n^{(i)}f_{ij}(1-f_{ij}) \text{for }i=l \\ 0 \text{ } \text{for }i\neq l \end{matrix}\right.$ (S3)

$\frac{dl^{2}(\theta)}{d\alpha_{i}d\gamma}=-\sum_{j=1}^{n^{(i)}} f_{ij}(1-f_{ij})Z_{ij}$ (S4)

$\frac{dl^{2}(\theta)}{d\gamma^{2}}=-\sum_{i=1}^{k} \sum_{j=1}^{n^{(i)}} f_{ij}(1-f_{ij})Z_{ij}Z_{ij}$ (S5)

Under null hypothesis *H*_0_ that *γ* = 0, we can obtain the maximum likelihood estimation (MLE) of *α_i_* and *f_ij_* as: $\hat{\alpha}_{i}=\log[\rho_{i}/(1-\rho_{i})]$ and $\hat{f}_{ij}=\rho_{i}$ with $\rho_{i}=n_{i}^{case}/n^{(i)}$. Such that we have, when *γ* = 0,

$\frac{dl(\theta)}{d\alpha_{i}}=0$ (S6)

$\frac{dl(\theta)}{d\gamma}=\sum_{i=1}^{k} 2n_{i}^{case}n_{i}^{control}(\hat{p}_{M}^{(i)case}-\hat{p}_{M}^{(i)control})/n^{(i)}$ (S7)

$\frac{dl^{2}(\theta)}{d\alpha_{i}d\alpha_{l}}=\left\{\begin{matrix} -n_{i}^{case}n_{i}^{control}/n^{(i)} \text{for }i=l \\ 0 \text{ } \text{for }i\neq l \end{matrix}\right.$ (S8)

$\frac{dl^{2}(\theta)}{d\alpha_{i}d\gamma}=-2n_{i}^{case}n_{i}^{control}\hat{p}_{M}^{(i)}/n^{(i)}$ (S9)

$\frac{dl^{2}(\theta)}{d\gamma^{2}}=-\sum_{i=1}^{k} 2n_{i}^{case}n_{i}^{control}\hat{p}_{M}^{(i)}(1+\hat{p}_{M}^{(i)})/n^{(i)}$ (S10)

where $\hat{p}_{M}^{(i)}$ is the allelic frequency of the SNP estimated from both cases and controls in the *i*th cohort, and the superscripts to $\hat{p}_{M}^{(i)}$ are used to distinguish allelic frequency estimated in cases from that in controls. Denote by *U*(*θ*) and *I*(*θ*) as the score and the information matrix respectively,

$U(\theta)=\left(\frac{dl(\theta)}{d\theta}\right)$ and $I(\theta)=-\left(\frac{dl^{2}(\theta)}{d\theta^{2}}\right)$ (S11)

The Score Test Statistic is $S=U_{\gamma}^{T}I_{\gamma}^{-1}U_{\gamma}$ , where

$U_{\gamma}=\sum_{i=1}^{k} 2n_{i}^{case}n_{i}^{control}(\hat{p}_{M}^{(i)case}-\hat{p}_{M}^{(i)control})/n^{(i)}$ (S12)

$I_{\gamma}=I(\theta)_{\gamma\gamma}-I(\theta)_{\gamma\alpha}I^{-1}(\theta)_{\alpha\alpha}I(\theta)_{\alpha\gamma}=\sum_{i=1}^{k} 2n_{i}^{case}n_{i}^{control}\hat{p}_{M}^{(i)}(1-\hat{p}_{M}^{(i)})/n^{(i)}$ (S13)

thus

$\begin{matrix} S=\frac{\left\{\sum_{i=1}^{k} 2n_{i}^{case}n_{i}^{control}(\hat{p}_{M}^{(i)case}-\hat{p}_{M}^{(i)control})/n^{(i)}\right\}^{2}}{\sum_{i=1}^{k} 2n_{i}^{case}n_{i}^{control}\hat{p}_{M}^{(i)}(1-\hat{p}_{M}^{(i)})/n^{(i)}} \\ =\frac{\left\{\Delta\hat{p}_{M}-\sum_{i=1}^{k} (r_{i}-s_{i})\hat{p}_{M}^{(i)}\right\}^{2}}{\sum_{i=1}^{k} 0.5\left\{\frac{n^{2}r_{i}s_{i}}{n^{(i)}\sum_{j=1}^{k} n_{j}^{case}\sum_{j=1}^{k} n_{j}^{control}}\hat{p}_{M}^{(i)}(1-\hat{p}_{M}^{(i)})\right\}} \end{matrix}$ (S14)

where notations $\Delta\hat{p}_{M}$, *s_i_* and *r_i_* are the same as those in equations (1), (8) and (11) in the main text. It is clear that Score Test Statistic for the logistic regression is identical to the Mantel-Haenszel Test Statistic as given by equation (11) in the main text.
