# Supplementary material for: A robust and efficient statistical method for genetic association studies using case and control samples from multiple cohorts
Source: BMC Genomics. 2013 Feb 8;14:88. doi: 10.1186/1471-2164-14-88 (PMC3626840; doi:10.1186/1471-2164-14-88)
Supplement: Additional file 6 — Predicting marker allele frequencies from control samples. [file 1471-2164-14-88-S6.doc]

**
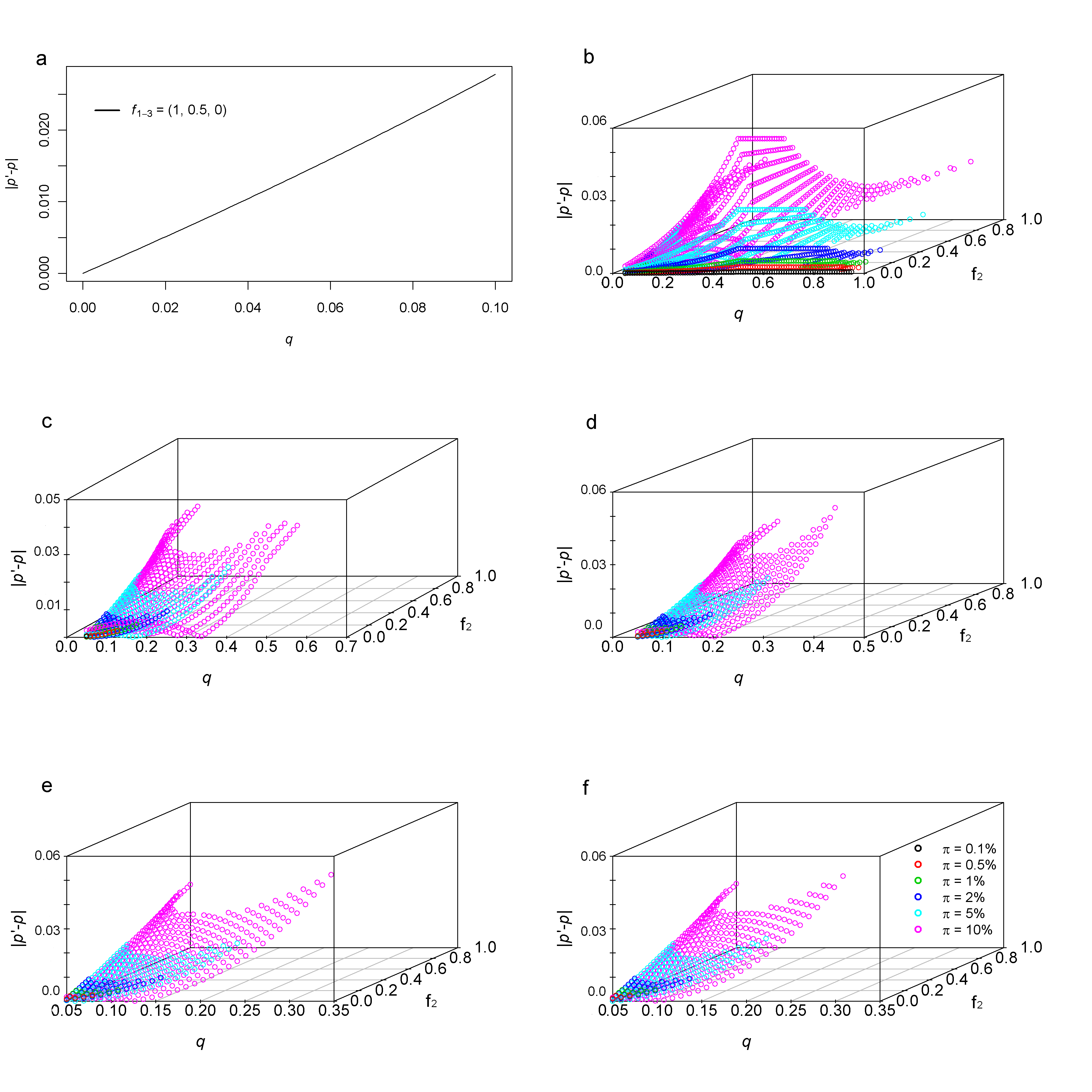
**

**Additional file 6** **Predicting marker allele frequencies from control samples.** Shown are the differences between *p*, the marker allele frequency in population, and *p’*, the expected marker allele frequency in a control sample under various disease prevalence and penetrance settings. (a) a full penetrance model in which *f*_1-3_ = (1, ½, 0) and *π* = *q* (disease allele frequency); (b-f) penetrance values for disease genotypes were determined from a given population prevalence *π* with a varying *f*_1_ = 0, 0.3, 0.5, 0.8 and 1. *p’* was calculated when the disequilibrium parameter *D* took its maximum value with marker allele frequency *p* = 0.5 and hence the bias presented here, i.e. *|p’ – p|*, was the maximum possible value for given frequency *q* and penetrance *f*_1-3_. In c-f, the *q* axis is truncated at *f*_3_ < 0 or *f*_3_ > 1 when *q* is large and/or very small.
