# Supplementary material for: A robust and efficient statistical method for genetic association studies using case and control samples from multiple cohorts
Source: BMC Genomics. 2013 Feb 8;14:88. doi: 10.1186/1471-2164-14-88 (PMC3626840; doi:10.1186/1471-2164-14-88)
Supplement: Additional file 7 — Simulation results from using biased estimates of maker allele frequency. [file 1471-2164-14-88-S7.doc]

**Additional file 7 Simulation results from using biased estimates of maker allele frequency**

| *p* | *q* | *D* | *D_max_* | *D_min_* | $\hat{p}$ | *χ*^2^ ± *s.d.* | ρ (%) |
| --- | --- | --- | --- | --- | --- | --- | --- |
| Pop. 1 | | | | | | | |
| 0.5 | 0.5 | 0 | 0.25 | -0.25 | 0.45 | 5.4±4.2 | 0.9 |
| 0.5 | 0.5 | 0 | 0.25 | -0.25 | 0.46 | 4.1±3.7 | 0.3 |
| 0.5 | 0.5 | 0 | 0.25 | -0.25 | 0.47 | 3.2±3.2 | 0.1 |
| 0.5 | 0.5 | 0 | 0.25 | -0.25 | 0.48 | 2.5±2.9 | 0.1 |
| 0.5 | 0.5 | 0 | 0.25 | -0.25 | 0.49 | 2.2±2.8 | 0 |
| 0.5 | 0.5 | 0 | 0.25 | -0.25 | 0.5 | 2.0±2.7 | 0 |
| 0.5 | 0.5 | 0 | 0.25 | -0.25 | 0.51 | 2.1±2.8 | 0 |
| 0.5 | 0.5 | 0 | 0.25 | -0.25 | 0.52 | 2.4±2.9 | 0 |
| 0.5 | 0.5 | 0 | 0.25 | -0.25 | 0.53 | 3.0±3.2 | 0.1 |
| 0.5 | 0.5 | 0 | 0.25 | -0.25 | 0.54 | 4.0±3.7 | 0.4 |
| 0.5 | 0.5 | 0 | 0.25 | -0.25 | 0.55 | 5.2±4.3 | 0.9 |
| Pop. 2 | | | | | | | |
| 0.3 | 0.7 | 0 | 0.09 | -0.21 | 0.25 | 6.4±4.7 | 1.3 |
| 0.3 | 0.7 | 0 | 0.09 | -0.21 | 0.26 | 4.7±4.0 | 0.5 |
| 0.3 | 0.7 | 0 | 0.09 | -0.21 | 0.27 | 3.5±3.4 | 0.3 |
| 0.3 | 0.7 | 0 | 0.09 | -0.21 | 0.28 | 2.7±3.0 | 0.3 |
| 0.3 | 0.7 | 0 | 0.09 | -0.21 | 0.29 | 2.2±2.8 | 0.2 |
| 0.3 | 0.7 | 0 | 0.09 | -0.21 | 0.3 | 2.0±2.7 | 0 |
| 0.3 | 0.7 | 0 | 0.09 | -0.21 | 0.31 | 2.1±2.7 | 0.1 |
| 0.3 | 0.7 | 0 | 0.09 | -0.21 | 0.32 | 2.4±2.9 | 0.1 |
| 0.3 | 0.7 | 0 | 0.09 | -0.21 | 0.33 | 3.2±3.2 | 0 |
| 0.3 | 0.7 | 0 | 0.09 | -0.21 | 0.34 | 4.2±3.7 | 0.3 |
| 0.3 | 0.7 | 0 | 0.09 | -0.21 | 0.35 | 5.6±4.4 | 1 |
| Pop. 3 | | | | | | | |
| 0.7 | 0.3 | 0 | 0.09 | -0.21 | 0.65 | 6.0±4.6 | 1 |
| 0.7 | 0.3 | 0 | 0.09 | -0.21 | 0.66 | 4.6±3.9 | 0.4 |
| 0.7 | 0.3 | 0 | 0.09 | -0.21 | 0.67 | 3.5±3.4 | 0.1 |
| 0.7 | 0.3 | 0 | 0.09 | -0.21 | 0.68 | 2.8±3.0 | 0.1 |
| 0.7 | 0.3 | 0 | 0.09 | -0.21 | 0.69 | 2.3±2.8 | 0 |
| 0.7 | 0.3 | 0 | 0.09 | -0.21 | 0.7 | 2.2±2.7 | 0 |
| 0.7 | 0.3 | 0 | 0.09 | -0.21 | 0.71 | 2.3±2.8 | 0 |
| 0.7 | 0.3 | 0 | 0.09 | -0.21 | 0.72 | 2.7±3.0 | 0 |
| 0.7 | 0.3 | 0 | 0.09 | -0.21 | 0.73 | 3.4±3.4 | 0 |
| 0.7 | 0.3 | 0 | 0.09 | -0.21 | 0.74 | 4.6±4.1 | 0.4 |
| 0.7 | 0.3 | 0 | 0.09 | -0.21 | 0.75 | 6.2±4.9 | 1.6 |
| Pop. 4 | | | | | | | |
| 0.5 | 0.5 | 0.15 | 0.25 | -0.25 | 0.45 | 167.5±42.1 | 100 |
| 0.5 | 0.5 | 0.15 | 0.25 | -0.25 | 0.46 | 173.7±42.4 | 100 |
| 0.5 | 0.5 | 0.15 | 0.25 | -0.25 | 0.47 | 178.5±42.6 | 100 |
| 0.5 | 0.5 | 0.15 | 0.25 | -0.25 | 0.48 | 181.8±42.8 | 100 |
| 0.5 | 0.5 | 0.15 | 0.25 | -0.25 | 0.49 | 183.7±42.9 | 100 |
| 0.5 | 0.5 | 0.15 | 0.25 | -0.25 | 0.5 | 184.4±42.8 | 100 |
| 0.5 | 0.5 | 0.15 | 0.25 | -0.25 | 0.51 | 183.8±42.7 | 100 |
| 0.5 | 0.5 | 0.15 | 0.25 | -0.25 | 0.52 | 181.8±42.5 | 100 |
| 0.5 | 0.5 | 0.15 | 0.25 | -0.25 | 0.53 | 178.4±42.3 | 100 |
| 0.5 | 0.5 | 0.15 | 0.25 | -0.25 | 0.54 | 173.4±42.2 | 100 |
| 0.5 | 0.5 | 0.15 | 0.25 | -0.25 | 0.55 | 165.9±42.0 | 100 |
| Pop. 5 | | | | | | | |
| 0.5 | 0.5 | 0.1 | 0.25 | -0.25 | 0.45 | 68.0±24.7 | 99.7 |
| 0.5 | 0.5 | 0.1 | 0.25 | -0.25 | 0.46 | 70.0±25.5 | 99.6 |
| 0.5 | 0.5 | 0.1 | 0.25 | -0.25 | 0.47 | 71.7±25.9 | 99.5 |
| 0.5 | 0.5 | 0.1 | 0.25 | -0.25 | 0.48 | 72.9±26.2 | 99.3 |
| 0.5 | 0.5 | 0.1 | 0.25 | -0.25 | 0.49 | 73.7±26.3 | 99.7 |
| 0.5 | 0.5 | 0.1 | 0.25 | -0.25 | 0.5 | 73.9±26.5 | 99.7 |
| 0.5 | 0.5 | 0.1 | 0.25 | -0.25 | 0.51 | 73.7±26.4 | 99.7 |
| 0.5 | 0.5 | 0.1 | 0.25 | -0.25 | 0.52 | 72.9±26.3 | 99.6 |
| 0.5 | 0.5 | 0.1 | 0.25 | -0.25 | 0.53 | 71.7±26.0 | 99.7 |
| 0.5 | 0.5 | 0.1 | 0.25 | -0.25 | 0.54 | 69.8±25.6 | 99.7 |
| 0.5 | 0.5 | 0.1 | 0.25 | -0.25 | 0.55 | 67.8±24.8 | 99.9 |
| Pop. 6 | | | | | | | |
| 0.5 | 0.5 | 0.05 | 0.25 | -0.25 | 0.45 | 20.4±10.4 | 45.6 |
| 0.5 | 0.5 | 0.05 | 0.25 | -0.25 | 0.46 | 19.1±10.7 | 39.3 |
| 0.5 | 0.5 | 0.05 | 0.25 | -0.25 | 0.47 | 18.4±11.3 | 36.4 |
| 0.5 | 0.5 | 0.05 | 0.25 | -0.25 | 0.48 | 18.2±11.7 | 36 |
| 0.5 | 0.5 | 0.05 | 0.25 | -0.25 | 0.49 | 18.2±11.9 | 36.5 |
| 0.5 | 0.5 | 0.05 | 0.25 | -0.25 | 0.5 | 18.1±12.0 | 36.8 |
| 0.5 | 0.5 | 0.05 | 0.25 | -0.25 | 0.51 | 18.2±11.9 | 36.7 |
| 0.5 | 0.5 | 0.05 | 0.25 | -0.25 | 0.52 | 18.2±11.6 | 36.8 |
| 0.5 | 0.5 | 0.05 | 0.25 | -0.25 | 0.53 | 18.5±11.1 | 36.9 |
| 0.5 | 0.5 | 0.05 | 0.25 | -0.25 | 0.54 | 19.1±10.6 | 39.7 |
| 0.5 | 0.5 | 0.05 | 0.25 | -0.25 | 0.55 | 20.4±10.1 | 45.9 |
| Pop. 7 | | | | | | | |
| 0.3 | 0.7 | 0.07 | 0.09 | -0.21 | 0.25 | 69.1±27.9 | 99.2 |
| 0.3 | 0.7 | 0.07 | 0.09 | -0.21 | 0.26 | 70.9±28.1 | 99.2 |
| 0.3 | 0.7 | 0.07 | 0.09 | -0.21 | 0.27 | 72.1±28.1 | 99.3 |
| 0.3 | 0.7 | 0.07 | 0.09 | -0.21 | 0.28 | 72.1±27.5 | 99.3 |
| 0.3 | 0.7 | 0.07 | 0.09 | -0.21 | 0.29 | 70.8±26.5 | 99.3 |
| 0.3 | 0.7 | 0.07 | 0.09 | -0.21 | 0.3 | 68.4±25.4 | 99.6 |
| 0.3 | 0.7 | 0.07 | 0.09 | -0.21 | 0.31 | 65.1±23.4 | 99.6 |
| 0.3 | 0.7 | 0.07 | 0.09 | -0.21 | 0.32 | 61.8±20.4 | 99.7 |
| 0.3 | 0.7 | 0.07 | 0.09 | -0.21 | 0.33 | 60.0±17.1 | 100 |
| 0.3 | 0.7 | 0.07 | 0.09 | -0.21 | 0.34 | 60.2±14.7 | 100 |
| 0.3 | 0.7 | 0.07 | 0.09 | -0.21 | 0.35 | 62.5±13.6 | 100 |
| Pop. 8 | | | | | | | |
| 0.3 | 0.7 | 0.05 | 0.09 | -0.21 | 0.25 | 31.5±16.3 | 73.6 |
| 0.3 | 0.7 | 0.05 | 0.09 | -0.21 | 0.26 | 32.0±17.1 | 74.1 |
| 0.3 | 0.7 | 0.05 | 0.09 | -0.21 | 0.27 | 32.6±17.5 | 75.2 |
| 0.3 | 0.7 | 0.05 | 0.09 | -0.21 | 0.28 | 33.1±17.8 | 76.4 |
| 0.3 | 0.7 | 0.05 | 0.09 | -0.21 | 0.29 | 33.3±17.8 | 76.9 |
| 0.3 | 0.7 | 0.05 | 0.09 | -0.21 | 0.3 | 33.2±17.6 | 77.3 |
| 0.3 | 0.7 | 0.05 | 0.09 | -0.21 | 0.31 | 33.0±16.9 | 78.5 |
| 0.3 | 0.7 | 0.05 | 0.09 | -0.21 | 0.32 | 33.0±15.7 | 81.3 |
| 0.3 | 0.7 | 0.05 | 0.09 | -0.21 | 0.33 | 33.4±14.2 | 85.5 |
| 0.3 | 0.7 | 0.05 | 0.09 | -0.21 | 0.34 | 34.8±12.7 | 90.9 |
| 0.3 | 0.7 | 0.05 | 0.09 | -0.21 | 0.35 | 37.3±11.9 | 94.8 |
| Pop. 9 | | | | | | | |
| 0.7 | 0.3 | -0.07 | 0.09 | -0.21 | 0.65 | 55.8±22.4 | 97.8 |
| 0.7 | 0.3 | -0.07 | 0.09 | -0.21 | 0.66 | 56.8±22.8 | 98.2 |
| 0.7 | 0.3 | -0.07 | 0.09 | -0.21 | 0.67 | 57.3±23.0 | 98.1 |
| 0.7 | 0.3 | -0.07 | 0.09 | -0.21 | 0.68 | 57.0±23.2 | 97.8 |
| 0.7 | 0.3 | -0.07 | 0.09 | -0.21 | 0.69 | 56.1±23.6 | 96.9 |
| 0.7 | 0.3 | -0.07 | 0.09 | -0.21 | 0.7 | 54.8±23.4 | 96.8 |
| 0.7 | 0.3 | -0.07 | 0.09 | -0.21 | 0.71 | 53.8±22.8 | 96.7 |
| 0.7 | 0.3 | -0.07 | 0.09 | -0.21 | 0.72 | 53.3±22.8 | 95.1 |
| 0.7 | 0.3 | -0.07 | 0.09 | -0.21 | 0.73 | 52.3±23.2 | 92.8 |
| 0.7 | 0.3 | -0.07 | 0.09 | -0.21 | 0.74 | 49.4±27.1 | 84 |
| 0.7 | 0.3 | -0.07 | 0.09 | -0.21 | 0.75 | 45.0±31.1 | 71.6 |
| Pop. 10 | | | | | | | |
| 0.7 | 0.3 | -0.05 | 0.09 | -0.21 | 0.65 | 27.9±15.1 | 68.2 |
| 0.7 | 0.3 | -0.05 | 0.09 | -0.21 | 0.66 | 28.5±15.3 | 68.5 |
| 0.7 | 0.3 | -0.05 | 0.09 | -0.21 | 0.67 | 28.8±15.3 | 69.1 |
| 0.7 | 0.3 | -0.05 | 0.09 | -0.21 | 0.68 | 28.8±15.4 | 68.8 |
| 0.7 | 0.3 | -0.05 | 0.09 | -0.21 | 0.69 | 28.2±15.4 | 67.4 |
| 0.7 | 0.3 | -0.05 | 0.09 | -0.21 | 0.7 | 27.8±15.6 | 66.1 |
| 0.7 | 0.3 | -0.05 | 0.09 | -0.21 | 0.71 | 26.9±15.8 | 66.3 |
| 0.7 | 0.3 | -0.05 | 0.09 | -0.21 | 0.72 | 26.2±16.3 | 65.4 |
| 0.7 | 0.3 | -0.05 | 0.09 | -0.21 | 0.73 | 23.9±17.6 | 61.5 |
| 0.7 | 0.3 | -0.05 | 0.09 | -0.21 | 0.74 | 21.5±19.5 | 54.3 |
| 0.7 | 0.3 | -0.05 | 0.09 | -0.21 | 0.75 | 17.4±20.4 | 39 |

Population genetic parameters defining case and control populations and statistical inference from present Method 1 by fitting with estimates of marker allele frequency $\hat{p}$ within ±0.05 from the true value. Means and standard deviations (s.d.) of χ^2^ test statistic were calculated from 1000 repeated samples of 200 cases and 200 controls under the additive genetic model. ρ (%) is the proportion in 1000 repeats in which the association test surpassed the Bonferroni threshold of P-value at 5×10^-5^. Population number is the same as in Table 3.
